# Malondialdehyde epitopes exposed on dying cells are potent modulators of complement activity and efferocytosis

**DOI:** 10.1016/j.jlr.2026.101100

**Published:** 2026-07-09

**Authors:** Nikolina Papac-Milicevic, Frida C. Mohlin, Mirlinda Ademi, Valentina Kovacic, Maria Górna, Kristina Djendjinovic, Clara J. Busch, David Weismann, Barbara Bartolini Gritti, Lejla Alic, Florentina Porsch, Daryna Katashynska, Laura Lopez Sans, Anna M. Blom, Christoph J. Binder

**Affiliations:** 1 Department of Laboratory Medicine, Medical University of Vienna, Vienna, Austria; 2 Department of Translational Medicine, Lund University, Malmö, Sweden; 3 Biological and Chemical Research Centre, Faculty of Chemistry, University of Warsaw, Warsaw, Poland

**Keywords:** Sterile inflammation, MDA-epitopes, complement system, efferocytosis

## Abstract

Multicellular organisms rely on efficient removal of dying cells to maintain tissue homeostasis. However, during severe tissue damage, when clearance mechanisms are overwhelmed, uncleared dying cells expose various danger-associated molecular patterns (DAMPs), such as malondialdehyde (MDA)- epitopes, which further propagate disease-associated sterile inflammation. MDA-epitopes are present on the surface of dying cells and are specifically recognized by several complement proteins, but the precise nature of their membrane exposure nor whether, and how, this interaction contributes to the removal of dead cells is unknown. Here, we demonstrate that MDA-epitopes were detected on the surfaces of dying cells generated by extrinsic or intrinsic triggers of apoptosis, ferroptosis, and necroptosis, and their occurrence was associated with loss of membrane integrity. Moreover, we show that MDA adducts activate the classical complement pathway by directly binding natural IgM antibodies and C1q and regulate the extent of complement activation through MDA-dependent preferential recruitment of the acute-phase variant of C4b-binding protein (C4BP (α6β0)). This MDA-epitope-guided and C4BP-tuned opsonization of dying cells with IgM, C1q, C4b, and C3b modulates their clearance by phagocytes in human and murine in vitro assays. Our findings identify MDA-epitopes as key mediators in recognizing and clearing dying cells, linking oxidative stress to immune activation and tissue homeostasis.

Cell death and efficient removal of dying cells are prerequisites for tissue morphogenesis and homeostasis (1, 2). In addition to apoptotic pathways, other types of programmed cell death (PCD), including necroptosis and ferroptosis have been identified (3). Once undergoing PCD, cells expose and/or release signals that alert and guide their clearance by professional and non-professional phagocytes. Prototypical examples of “eat and find me” signals, which are recognized by phagocyte receptors as well as complement components include triphosphate nucleotides (ATP, UTP), phosphatidylserine (PtdSer), oxidized phospholipids, lysophosphatidylcholine, and fractalkine (CX_3_CL1) (4, 5, 6, 7, 8). Apoptotic cells are usually cleared before their membrane integrity is lost, and both apoptotic cells and the efferocytosing phagocytes produce and secrete tolerogenic signals to ensure a balanced reaction of the immune system. Defective efferocytosis leads to the release of damage-associated molecular patterns (DAMPs) from dying cells, resulting in impaired self-tolerance and sterile inflammation, which is a pathological driver of many chronic inflammatory diseases, including atherosclerosis (1).

We have previously characterized oxidation-specific epitopes (OSEs) as a class of DAMPs representing key targets of innate immune responses that trigger inflammation in atherosclerosis. OSEs are post-translational modifications that are generated when membrane phospholipids are oxidized, giving rise to the formation of aldehyde adducts on various biomolecules. They have been documented on apoptotic cells, damaged structures such as oxidized lipoproteins and on a subset of extracellular vesicles (4, 9, 10, 11, 12, 13, 14). Malondialdehyde (MDA)-derived epitopes, which include the immunogenic MDA-haptens itself and its immunodominant form malondialdehyde–acetaldehyde (MAA) adducts, represent major examples of OSEs (15, 16). Like other DAMPs, they are non-infectious pro-inflammatory mediators recognized by various cellular and humoral components of innate immunity (4, 9, 13, 14, 15, 17, 18, 19, 20, 21, 22, 23). Scavenger receptors, natural IgM antibodies, complement factor H (FH) and FH-related proteins 1, 3 and 5 (FHR1, FHR3 and FHR5) have been demonstrated to bind MDA-epitopes and are involved in their neutralization and removal (4, 9, 10, 11, 14, 18, 19, 20, 22, 24). An imbalance between the generation of MDA-epitopes and their elimination leads to their accumulation, which triggers sterile inflammation in atherosclerosis, age-related macular degeneration, or steatohepatitis development emphasizing the importance of efficient recognition and clearance of OSE by components of innate immunity (19, 24, 25).

However, neither the time-resolved occurrence of MDA-epitopes on dying cells nor their role in guiding homeostatic functions of innate immunity are known. Here, we show that MDA-epitopes are exposed on the surface of dying cells with compromised membrane integrity undergoing extrinsic and intrinsic apoptosis pathways, ferroptosis, and necroptosis. We could demonstrate that these MDA-modified proteins not only initiate classical complement activation through direct engagement of natural IgM and C1q but also constrain excessive complement activity before the propagation stage via selective recruitment of the acute-phase isoform of the complement regulator C4b-binding protein (C4BP (α6β0)). Functionally, this balanced MDA-dependent complement activation promotes targeted opsonization with IgM, C1q, C4b, and C3b, thereby fine-tuning the recognition and uptake of dying cells by efferocytic phagocytes.

Thus, our data identify MDA adducts as important marks of cellular waste that play a significant role in maintaining tissue homeostasis, particularly in situations where the clearance of cells undergoing apoptosis fails.

## Materials and Methods

### OSEs, proteins, cells, and sera

Malondialdehyde-modified bovine serum albumin (MDA-BSA), malondialdehyde-acetaldehyde-modified BSA (MAA-BSA), 4-hydroxynonenal-modified BSA (4-HNE-BSA), copper sulfate–oxidized low-density lipoprotein (CuOx-LDL), malondialdehyde-acetaldehyde-modified LDL (MAA-LDL) and malondialdehyde-modified LDL (MDA-LDL) were generated and validated as previously described (9). Phosphorylcholine-modified BSA (PC-BSA) was purchased from Biosearch Technologies.

Jurkat T cells, THP-1, HT-1080, RAW 264.7, and HeLa were purchased from ATCC® (ATCC®). FADD−/− Jurkat T cells were a kind gift from Dr Manuele Rebsamen (University of Lausanne). Shortly, Jurkat T cells, THP-1, and FADD−/− Jurkats were grown in Gibco™ RPMI-1640 GlutaMax™ medium (Life Technologies), and HT-1080, RAW264.7 and HeLa were grown in GIBCO™ Dulbecco's Modified Eagle (DMEM) GlutaMAX™ Medium (Life Technologies). Media were supplemented with 100 units of penicillin per ml plus 100 μg of streptomycin per ml (Sigma-Aldrich) in addition to 10% fetal bovine serum. Primary thymocytes were isolated from female 8-week-old C57BL/6 mice. After surgical removal, the mouse thymus was dissected in 1% BSA in PBS (1%BSA/PBS), disrupted by pipetting, and passed through a 70 μm nylon strainer (Corning). Released cells were counted and maintained in the RPMI-1640 medium. All used cells were grown in an incubator with 95% humidity, 5% CO2, and 37°C.

To study the appearance of MDA-epitopes we used two well-characterized monoclonal anti-MDA IgM antibodies, LR04 and NA17. Both antibodies were cloned from murine spleens and selected for binding to MDA-modified low-density lipoprotein (MDA-LDL), shown to bind MDA-epitopes on apoptotic cells and to mediate uptake by macrophages or inhibit pro-inflammatory effects of MDA (10, 24, 26, 27, 28).

Human C1q and C4BP were purified from plasma, as previously described (29, 30). Recombinant wild-type C4BP and different mutants lacking individual CCP domains were expressed in HEK (293) cells and purified as described (31, 32). Recombinant FI was expressed recombinantly and purified as described previously (32). Plasma-purified FH and FB were obtained from Complement Technology Inc. C4b was purchased from Complement Technology and radiolabeled with 125I using the chloramine T method (33). The specific activity was 0.4–0.5 MBq/μg of protein. C4met and C3met are C4b- and C3b-like molecules that were prepared by incubating C4 and C3 (both purified from plasma as described previously (34)) with 100 mM methylamine, pH 8.0, for one hour at 37°C, to hydrolyze the internal thioester bond. After incubation, the proteins were dialyzed extensively against Tris-buffered saline (TBS).

Normal human serum (NHS) was obtained from healthy volunteers (N = 5) under the ethics approval EK1845/2015 given by the Ethics Committee of the Medical University of Vienna. Following blood drawing, blood was allowed to clot for 30 min at room temperature (RT) using Z serum Sep Clot Activator tubes (Greiner Bio-one, Kremsmünster, Austria), centrifuged for 30 min at 4°C at 2000g, and the collected serum was snap frozen and then stored at −80°C. Heat-inactivated NHS (HI-NHS) was prepared by incubating NHS for 30 min at 56°C. C1q-depleted serum was purchased from Quidel and factor B-depleted serum from Complement Technology Inc.

Serum from 8 to 10 weeks old male *C4bp+/+* and *C4bp−/−* littermate animals was collected into MiniCollect Z Serum Sep tubes (Greiner Bio-One) and processed as above.

### Animals

All mice were bred and housed in a barrier-specific pathogen-free breeding facility at the Department of Biomedical Research, Medical University of Vienna, Austria. C4BP-deficient animals were previously generated by Rick A. Wetsel at The University of Texas (35). *C4bp−/−* mice were backcrossed to the C57BL/6J Himberg background. The animals were kept in individually ventilated cages under a 12-h light/dark cycle, with ad libitum access to food and water. Experiments were conducted using age- and sex-matched adult littermates (i.e., 8 weeks of age or older). All experimental protocols using mice were approved by the animal experimentation committee of the Medical University of Vienna and the Austrian Ministry of Sciences.

### Induction of apoptosis and other programmed cell death pathways

Apoptosis was induced by UVC irradiation (0.5 mJ/cm^2^, 5 mJ/cm^2^, 25 mJ/cm^2^, and 100 mJ/cm^2^)2 μM staurosporine (Sigma-Aldrich), or 1 μg/ml of anti-CD95 antibody for 16 h of Jurkat T cells and RAW 264.7 murine macrophages. In case of primary thymocytes, a time course experiment was performed with irradiation at 100 mJ/cm^2^ for different timepoints. When required, control cells were treated with dimethyl sulfoxide (DMSO) or 1 μg/ml isotype control IgG1 antibody (BD Pharmigen). Following apoptosis induction, cells were kept in the incubator in serum-free RPMI-1640 media supplemented with 50 μg/ml of BSA. Ferroptosis was induced in HT-1080 cells with 10 μM erastin (Sigma-Aldrich) for 16 h in serum-free DMEM media with 50 μg/ml BSA. DMSO was used as a vehicle control, and an inhibitor of ferroptosis, ferrostatin, was applied at a concentration of 1 μM (Sigma-Aldrich). Necroptosis was induced in FADD−/− Jurkat T cells with 10 ng/ml of human TNF (R&D Systems, Bio-Techne) for 16 h in serum-free RPMI-1640 with 50 μg/ml of BSA. For inhibition of necroptosis, 1 μM of necrostatin-1 (Nec-1s) (Merck) or 1 μM of necrosulfonamide (NSA) (Merck) was added.

Cell death and viability were assessed by flow cytometry using Annexin V apoptosis detection PE or FITC kit (eBiosciences), and by 7-Aminoactinomycin (7AAD) staining (eBiosciences) according to the manufacturer’s protocol. Samples were analyzed using a BD FACS Calibur or LSR Fortessa (BD Biosciences) flow cytometer.

### Detection of MDA/MAA-epitopes on dying cells by flow cytometry assay

To demonstrate the appearance of MDA-epitopes, after blocking with 1% BSA/PBS, 300,000–500,000 of dying cells/well were stained with murine MDA/MAA-specific IgM antibodies that were previously generated (10, 14). MDA/MAA-specific IgMs, LR04, NA17, and mouse isotype control IgM (BioLegend) were used to stain cells at 10 μg/ml in 100 μl of 1% BSA/PBS buffer for 20 min at 4°C. The signals for MDA/MAA-epitopes were detected with 0.2 μg/ml of secondary goat anti-mouse IgM antibody conjugated with allophycocyanin (APC) (eBiosciences). Finally, analysis was performed using a FACS Calibur (BD Biosciences).

### Confocal microscopy

The presence and subcellular localization of MDA-epitopes on dying cells were evaluated by immunocytochemistry. In brief, 500,000 Jurkat T cells or FADD−/− Jurkat T cells were seeded in 12-well plates, and cell death was induced. Similarly, HT-1080 cells were grown to 80% confluence in 4-well chamber slides (Nalge Nunc International), and ferroptosis was induced. After 16 h dead cells were blocked with 1% BSA/PBS for 20 min at 4°C. If required, before primary antibody staining, cells were stained with WGA-633 (5 mg/ml) in HBSS for 10 min at 37°C. Cells were stained with 10 μg/ml of monoclonal mouse anti-MDA IgM antibodies (LR04, NA17) or an isotype control (BioLegend), followed by anti-mouse IgM secondary antibodies conjugated to Alexa fluor 488 at a 1:300 dilution (Molecular Probes, USA). 4′,6-diamidino-2-phenylindole (DAPI) (Sigma-Aldrich) was used to stain nuclei. If required, stained cells were transferred to glass slides (Corning). Fluorescence microscopy was performed using an inverted point laser scanning confocal microscope (LSM-780, LSM-700, Zeiss, Jena, Germany). Confocal images acquired were analyzed using the ZEN 2012 black software (Zeiss).

### Complement activation assays

Complement activation for all three complement pathways was assessed using microtiter plate-based assays. If not stated otherwise, all incubation steps were done at a volume of 50 μl at RT. Microtiter plates were coated with 2.5 μg/ml MAA-BSA in coating buffer (75 mM sodium carbonate, pH 9.6), together with negative BSA controls (untreated and sham-treated BSA) and positive controls (2.5 μg/ml heat-aggregated IgG (Kiovig, Takeda Pharmaceuticals) for the classical pathway, 100 μg/ml mannan (Sigma-Aldrich) for the lectin pathway and 20 μg/ml zymosan (Sigma-Aldrich) for the alternative pathway. After each incubation step, wells were washed extensively with 50 mM Tris-HCl, pH 8.0, 150 mM NaCl and 0.1% Tween 20. The plates were blocked using 250 μl of 1% BSA/PBS. Pools of NHS or HI-NHS were diluted in either GVB++ (5 mM veronal buffer, pH 7.3, containing 150 mM NaCl, 0.1% gelatin, 1 mM MgCl2, and 0.15 mM CaCl2) for the classical and lectin pathways or MgEGTA (2.5 mM veronal buffer, pH 7.3, containing 70 mM NaCl, 140 mM glucose, 0.1% gelatin, 7 mM MgCl2 and 10 mM EGTA) for the alternative pathway and incubated on the plate for 20–60 min at 37°C. Complement activation was detected using specific antibodies against C1q (Dako, Agilent Technologies), C4c (Dako), C3d (Dako), C9 (Complement Technology Inc.), MBL (R&D Systems) and factor B (Quidel), followed by horseradish peroxidase-conjugated secondary antibodies (Dako). Assays were developed with an OPD substrate (Dako) and absorbance measured at 490 nm. All antibodies were diluted in 1% BSA/PBS.

### Direct binding assays (ELISAs)

The binding assays of C1q, C3b, and C4BP to different OSE were performed in microtiter plates. All incubation steps were done at a volume of 50 μl, if not stated otherwise. After each incubation step, the wells were washed extensively as described for the complement activation assays. Different OSEs (either OSE-modified BSA or LDL) were coated at a concentration of 2 μg/ml in PBS overnight at +4°C, and wells were blocked with 1% BSA/PBS. C1q, C3b (i.e., C3met), plasma-purified (α7β1+protein S) or recombinant wild-type C4BPs (α6β0), and deletion mutants lacking individual CCP domains were added to the plate at increasing concentrations in 50 mM HEPES, 100 mM NaCl, 2 mM CaCl2, pH 7.4 and incubated for 1 h at 37°C. Binding was detected using specific antibodies against C1q (Dako), C4BP (PK9008, prepared in-house), and C3c (Dako), followed by horseradish peroxidase-conjugated secondary antibodies (Dako), and developed and measured as described above.

For determining the nature of C4BP binding to MDA/MAA epitopes 1 μg/ml of MAA-BSA or sham-treated BSA in PBS were coated to 96-well white or round-bottomed microtiter plates overnight at +4°C. Plates were then washed with PBS and blocked for one hour at room temperature (RT) with 1% BSA/PBS. Afterwards, they were incubated for 2 h at RT with 1% BSA/PBS with plasma purified C4BP (α7β1+PS) or recombinant C4BP (α6β0) (1 μg/ml for NaCl or 1.6 μM) with or without increasing concentrations of NaCl. Following another washing step, bound C4BP was detected using a rabbit anti-C4BP antibody (generated in-house) at a concentration of 0.5 μg/ml in 1% BSA/PBS and incubated for 2 h at RT. Following, a secondary anti-rabbit-IgG-alkaline phosphatase-labeled antibody (Sigma-Aldrich) (1:20,000 in 1% BSA/PBS) for 2 h at RT. Detection of C4BP was performed by chemiluminescence using a 33% solution in water of LumiPhos for 1 h at RT and in the dark (Lumigen). Plates were read on a Synergy 2 plate reader (BioTek). Data are expressed as relative light units (RLU) per 100 ms.

For detection of mouse C4BP binding to MDA/MAA epitopes, 96-well white or round-bottomed microtiter plates were coated with 1 μg/ml of MAA-BSA or sham-treated BSA in PBS at +4°C overnight. Coated plates were then washed with PBS and blocked for 1 h at RT with 1% BSA/PBS. Afterwards, they were incubated for 2 h at RT with 1%BSA/PBS containing dilutions of 0.01%, 0.1%, 0.5%, 1.5%–10% plasma from 10-week-old male C57BL/6J Himberg mice. Bound murine C4BP (mC4BP) was detected using a rabbit anti-mouse C4BP antibody (generated in-house) at a concentration of 1.2 μg/ml in 1% BSA/PBS and incubated for 2 h at RT. After another washing step, a secondary anti-rabbit-IgG-alkaline phosphatase-labeled antibody (Sigma-Aldrich) was added (1:20,000 in 1% BSA/PBS) and incubated at RT for 2 h. Detection of bound mC4BP was performed by chemiluminescence as described above. Data are expressed as relative light units (RLU) per 100 ms.

### C4b degradation assays

To determine C4BP’s cofactor activity to factor I in C4b degradation when bound to MDA/MAA-epitopes, two degradation assays were performed: one in solution and one on a plate. In the first assay, recombinant C4BP (α6β0) was pre-incubated with MAA-BSA (or negative controls), in a volume of 20 μl, before adding C4met, FI, and ^125^I -labeled C4b to a total volume of 40 μl. C4BP, at a concentration of 30 μg/ml, was pre-incubated with 0–2 μM of the different BSA variants (0–132 μg/ml) or MK104 (0–300 μg/ml) (a monoclonal antibody against C4BP, prepared in-house and known to inhibit C4BP’s cofactor activity) as a positive control, in TBS for 2 h at RT. After the pre-incubation, 50 μg/ml C4met, 8 μg/ml recombinant FI, and trace amounts of ^125^I -labeled C4b were added to the mixture to a final volume of 40 μl and incubated further for 1.5 h at 37°C. The reactions were terminated by the addition of SDS-PAGE sample buffer containing 25 mM dithiothreitol (DTT), heated at 95°C for 3 min, and applied on a 10%–15% gradient SDS-PAGE gel. The amount of C4b degradation was quantified using a Typhoon FLA 9500 scanner (GE Healthcare) and analyzed with the ImageGauge software (FujiFilm, Tokyo, Japan). Data are shown as a ratio between the intensity of the C4d degradation fragment and the intact α-chain of C4b.

In the second degradation assay, C4BP was bound to plate-immobilized MAA-BSA (or negative controls). Microtiter plates were coated with 10 μg/ml MAA-BSA (or negative controls) in a volume of 100 μl in PBS overnight at 4°C and then blocked with 250 μl of 1% BSA in TBS for 1 h at 37°C. Recombinant C4BP (α6β0) was diluted to 0–200 μg/ml in a volume of 100 μl TBS and incubated on the plate for 7 h at RT. After washing, 50 μg/ml recombinant FI and trace amounts of ^125^I -labeled C4b were added to the plate in a volume of 50 μl in TBS and incubated overnight at 37°C. The reactions were terminated directly (without washing the plate) by adding SDS-PAGE sample buffer containing 25 mM DTT to the plate. The mixture was then transferred to a tube, heated for 3 min at 95°C, and the degradation of C4b was then detected as described above.

### Surface plasmon resonance (SPR) and kinetics analysis

MAA-BSA diluted in sodium acetate (pH 4.0) was immobilized on a CM5 sensor chip using the amine coupling reagent kit (GE Healthcare) at 25°C. The carboxymethyl dextran surface was activated with EDC/NHS solution. As a reference, the same procedure was performed on another flow cell without the protein (blank immobilization). Moreover, the remaining activated groups were blocked with 1M ethanolamine. PBS (Sigma-Aldrich) was used as a running buffer. Various C4BP variants were diluted in running buffer at 100, 50, 25, 12.5, and 6.25 nM and injected at a flow of 30 μl/min for 120 s (association) over the immobilized MAA-BSA and blank reference. A Biacore X100 (GE Healthcare) was used for SPR detection. Dissociation was monitored for 600 s, and regeneration was performed with 2.5 M NaCl for 25 s at 30 μl/min. Lacking insights on the number of CCP7 domains per one C4BP that can be involved in MDA/MAA binding, kinetic constants were calculated by the global fitting method, assuming the most basic 1:1 mode (1:1 Langmuir binding model).

### Collection of MDA^high^ (H) and MDA^low^ (L) cellular fractions

Jurkat T cells or RAW 264.7 were killed by UVC 100 mJ/cm2 irradiation, and apoptotic cells were maintained, as described above. Dying cells were incubated with 40 μg/ml of either isotype control or the MDA-specific LR04 antibody for 30 min at +4°C, washed in PBS, and then incubated with anti-mouse IgM microbeads (Militenyi Biotech, Cologne, Germany). Labeled cells were then passed through a MS MACS column (Miltenyi Biotech), and flow through containing MDA^high^ (H) (from isotype control-treated cells) and MDA^low^ (L) (from LR04-treated cells) fractions were collected according to the manufacturer's protocol. The efficiency of fractionation was confirmed using flow cytometry after staining an aliquot of cells with 10 μg/ml of isotype control or LR04 antibodies, which was detected with an anti-IgM APC (eBiosciences) and an Annexin V Apoptosis Detection Kit (eBiosciences).

### Binding of opsonins and C4BP to the surface of dying cells (flow cytometry assays and competitive flow cytometry assay)

Twenty μg/ml of recombinant C4BP proteins in 1% BSA/binding buffer (10 mM HEPES, 150 mM NaCl, 5 mM KCl, 1 mM MgCl2, 1.8 mM CaCl_2_) or various mixtures of binding buffer containing 0.5%, 1% or 2% human serum were added to MDA^high^ (H) and MDA^low^ (L) fractions of apoptotic cells and incubated for 2 h at +4°C. After washing, bound proteins were detected using specific antibodies in 1%BSA/binding buffer for 20 min at 4° in the dark. Human C4BP was detected using 1 μg/ml of PK9008 (made in-house), C1q by 1 μg/ml of anti-C1q antibody (Cedarlane), IgM with 0.25 μg/ml of anti-mouse IgM (eBiosciences), and C3c with 10 μg/ml of a FITC-labeled anti-C3 antibody (Dako). When required, a secondary antibody conjugated to APC or AF680 was used at a 1:200 dilution for 30 min at 4°C.

Three hundred thousand viable or apoptotic RAW264.7 and Jurkat T cells were incubated in 100 μl binding buffer with 0%, 2.5% or 5% normal serum of male *C4bp+/+* or *C4bp−/−* C57BL/6J mice for 40 min at 4°C. Afterward, bound mC4BP was detected with 2.1 μg/ml of a rabbit anti-mouse C4BP antibody (made in house), incubated for 20 min at 4°C, followed by a secondary antibody conjugated to AF680 at a 1:200 dilution and incubated for 30 min at 4°C.

Flow cytometry was used to monitor the binding of mC4BP to apoptotic cells in the presence or absence of increasing concentrations of MAA-BSA or sham BSA control. Sixteen hours post-irradiation, 350,000 cells were incubated in 2.5% of normal murine serum (NMS) in PBS with increasing concentrations of MAA-BSA or sham BSA ranging from 0.5 to 10 μg/ml, for 40 min at 4°C. Afterward, bound mC4BP was detected, as described above. For each experiment, triplicate determinations were done, and the percentage of apoptotic cells bound by mC4BP was determined. The obtained data are calculated as the ratio of mC4BP-bound cells for a particular concentration of competitor (B) and the binding where no competitor was added (B0).

In all opsonization experiments, cell viability was monitored, as mentioned previously. Finally, analysis was performed using a FASC Calibur or LSR Fortessa flow cytometer (BD Biosciences).

### Efferocytosis assay

Efferocytosis assays were done with human and murine cells. To generate THP1-like macrophages, 250,000–300,000 THP1 cells were seeded per well of 24-well plate. Seeded cells were treated with 100 nM PMA in complete RPMI medium for 48 h. Subsequently, cells were washed with RPMI and allowed to recover for 24 h in complete RPMI medium before performing the efferocytosis assay. To obtain primary macrophages, 2 ml of 3% thioglycolate medium was injected into the peritoneum of male *C4bp+/+* mice. Seventy-two hours post-injection, mice were sacrificed by cervical dislocation, and a peritoneal lavage was performed with 10 ml of sterile Hank’s Balanced Salt solution. The number of macrophages collected by peritoneal lavage was determined using a CASY cell counter (OMNI Life Science, Bremen, Germany) and seeded at 250,000 cells/well in a 24-well plate in RPMI media supplemented with 2.5% FBS. The next day, MDA^high^ (H) and MDA^low^ (L) fractions of UVC-irradiated and apoptotic RAW 264.7 cells were collected and stained with pHrodo (Thermo Fisher Scientific) in PBS for 30 min at 37°C. Dead MDA^high^ (H) and MDA^low^ (L) cells were fed to PMA matured THP1 macrophage-like cells to thioglycolate elicited macrophages in serum-free RPMI in the absence or presence of 5% of normal or heat-inactivated NHS, *C4bp+/+* or *C4bp−/−* serums for 1 h at 37°C in ratio 0.8–1:1. At the end of the experiment, cells were washed 2x in ice-cold PBS and scraped.

For flow cytometry analysis, cells were transferred to a V-bottom plate and incubated with 100 μl of 2.5 μg/ml anti-CD16/32 antibody (Clone 93, Thermo Fisher Scientific) for 20 min at 4°C to block Fcγ receptors. After washing, THP1 macrophages and primary murine macrophages in 1% BSA/PBS, were stained for 20 min at 4°C with an antibody cocktail containing anti-CD11b FITC (clone M1/70, Thermo Fisher Scientific, human and mouse efferocytosis experiment) at 1:200 and anti-F4/80 PE-Cy7 (clone BM8, BioLegend, only mouse experiment) at 1:800 in 1% BSA/PBS. Final analysis was performed using an LSR Fortessa flow cytometer (BD Biosciences).

### Statistical analysis

All data were generated with at least two independent experiments and triplicate determinations. Statistical analysis of data was performed using GraphPad Prism software, version 9 (GraphPad Software Inc.). Data are represented as mean ± SD (n > 3). Two groups were compared using a paired (when applicable) or unpaired Student’s *t* test. One-way or two-way ANOVA followed by Tukey‘s or Bonferroni's multiple comparison, was used for comparing three or more groups. Statistical analysis with *P* < 0.05 was considered as statistically significant.

## Results

### The surface exposure of MDA-epitopes on dying cells is associated with loss of membrane integrity

**Fig. 1:**
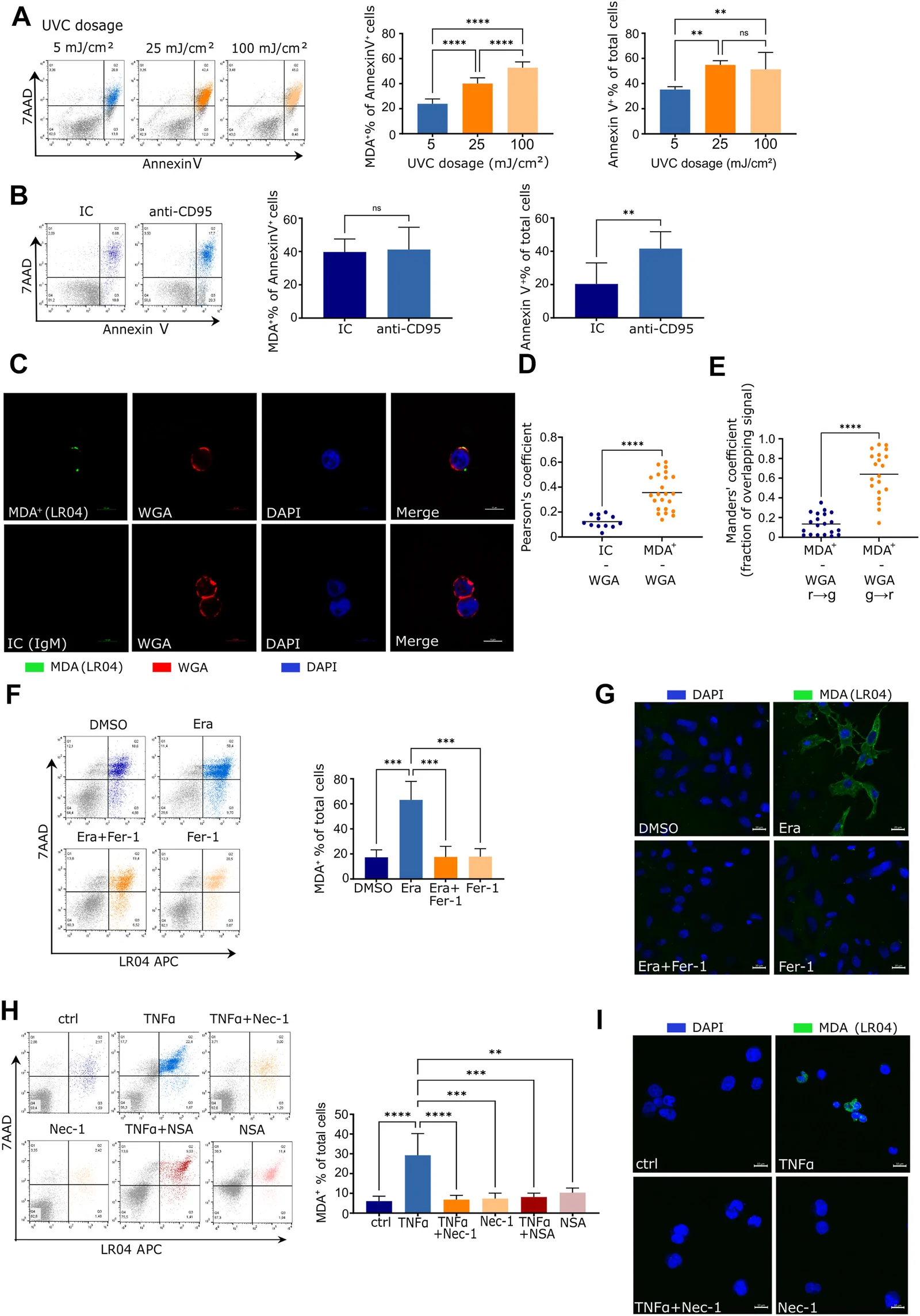
MDA-epitopes are generated on apoptotic cells independent of the apoptosis inducer and appear on the surface of damaged cellular membranes. A, B: Flow cytometry assays for determining the presence of MDA-epitopes on the surface of apoptotic cells. Representative viability plots are divided into quadrants based on 7AAD and Annexin V positivity, while MDA^+^ cells are distinguished by color (left panel). Quantifications of MDA^+^ within Annexin V^+^ (middle panel) and Annexin V^+^ populations within total cells are shown (right panel). A.) Presence of MDA-epitopes on Jurkat T cells upon increasing UVC irradiation energy dose (5, 25 and 100 mJ/cm^2^, N = 3 independent experiments with biological duplicates or triplicates). B: Induction of extrinsic apoptotic pathway by anti-CD95 or control isotype antibody (IC) and appearance of MDA-epitopes on Jurkat T cells (N = 4 independent experiments with one value per experiment). C: Colocalization of MDA-epitopes (LR04 = green, first upper row) with cellular membrane (WGA = red, second column in images) by confocal microscopy. Nucleus was counterstained with DAPI (blue, third column in images). Yellow fluorescence reveals the colocalization of MDA^+^ signal (low to middle abundance) and cellular membrane (fourth column-merged image). Staining of apoptotic cells with IC IgM, DAPI and WGA (negative control, lower row) (scale bar represents 10 μm, LSM-700, objective 63x, N = 2 independent experiments, IC = 10 images per experiment, LR04 = 20 images per experiment) D: Colocalization analysis based on Pearson's correlation between IC or MDA^+^ signal and cellular membrane (WGA). Each spot represents a single analyzed image. E: Colocalization analysis based on Manders' coefficient, fraction of overlap between two channels, within MDA^+^ images. Data are represented as means. Each spot represents a single analyzed image. F and H: Flow cytometry assays for determining the presence of MDA-epitopes on ferroptotic and necroptotic cells. Representative plots divided into quadrants according to 7AAD and MDA positivity (LR04) (left panel) and corresponding quantifications (right panel) are shown (four independent experiments with single or duplicate values). G and I: Confocal images of MDA-epitopes. F) Detection of MDA-epitopes on ferroptotic HT1080 cells. Ferroptosis was induced using erastin and inhibited by Ferrostatin-1 (Fer-1). G) Confocal images of MDA-epitopes (LR04 = green, DAPI = blue) on ferroptotic HT1080 cells (scale bar represents 20 μm, LSM780, objective 20x). H: Detection of MDA-epitopes on the surface of necroptotic FADD−/− Jurkat cells. Necroptosis was induced by TNF and inhibited by Necrostatin-1 (Nec-1) or by Necrosulfonamide (NSA) (N = 6 independent experiments with single value). D: Confocal images of MDA-epitopes (LR04 = green) on necroptotic FADD−/− Jurkat cells (DAPI = blue) (scale bar represents 10 μm, LSM-780, objective 40x). Bars represent mean ± SD values. Statistical differences were tested by Student's *t* test, one-way ANOVA, with Tukey’s multiple comparison (∗∗*P*-value ≤ 0.01, ∗∗∗*P*-value ≤ 0.001, ∗∗∗∗*P*-value ≤ 0.0001, ns = not significant).

**Supplemental Figure 1:**
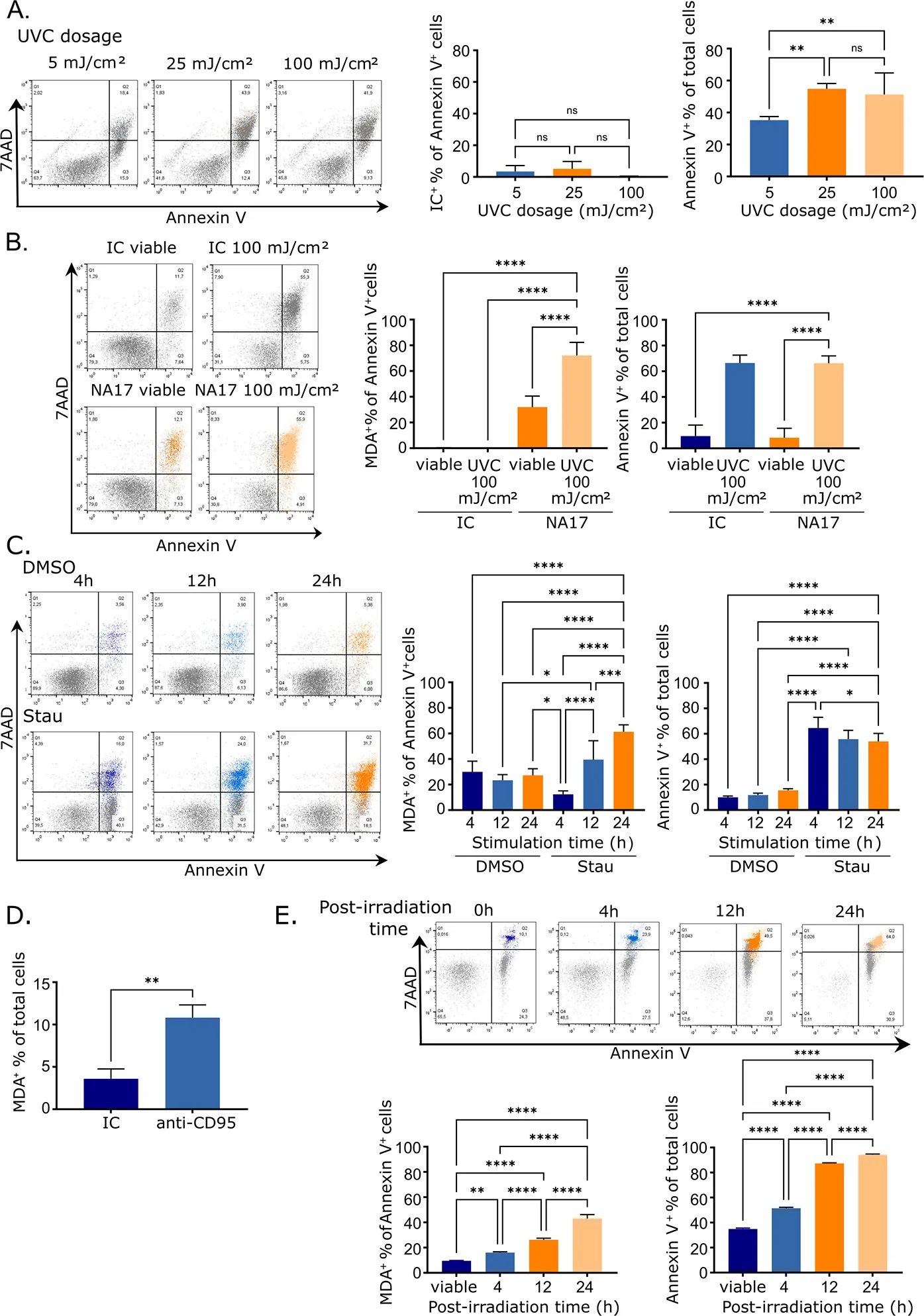


MDA-epitopes have been documented on apoptotic as well as necrotic cells, but the molecular requirements for their surface exposure nor their association with specific PCD pathways are known (10, 11, 19). We characterized the occurrence of MDA-epitopes on apoptotic Jurkat T cells by flow cytometry using previously characterized natural IgM antibodies specific for MDA-epitopes, particularly for MAA-structures (15, 26). UVC irradiation increased MDA-antibody binding (LR04) in a dose-dependent manner (Fig. 1A, right and middle panel) while an isotype control antibody showed no binding (Supplemental Fig. S1A). Co-staining with Annexin V for PtdSer exposure and 7-Aminoactinomycin D (7AAD) for loss of membrane integrity revealed that most MDA-epitopes localized to late apoptotic cells with disrupted cellular membranes (7AAD^+^, Annexin V^+^, ∼ 85%) (Fig. 1A, right upper quadrants within left panel), whit few on early apoptotic cells (7AAD^−^, Annexin V^+^) and negligible amounts on viable cells (7AAD^−^, Annexin V^−^). Similar findings were obtained using another anti-MDA-epitope IgM antibody (NA17) (Supplemental Fig. S1B).

To determine if the appearance of MDA-epitopes depends on apoptosis inducers, Jurkat T cells were treated with staurosporine. Similarly to UVC irradiation, MDA-epitopes were scarce on early apoptotic cells but abundant on late apoptotic cells across all time points (Supplemental Fig. S1C, lower panels), while control DMSO treatment had no effect on their appearance (Supplemental Fig. S1C, top panels).

Irradiation with UVC and staurosporine induce intrinsic apoptosis via irreparable cellular stress. To test whether the extrinsic apoptosis pathway would result in the exposure of MDA-epitopes, Jurkat T cells were treated with anti-CD95 antibody for 16h. Although the apoptosis extent was less pronounced than with intrinsic inducers, the percentage of MDA^+^ apoptotic cells relative to total cells increased (Figs. 1B, Supplemental Fig. S1D), demonstrating the most epitopes on late apoptotic cells (Fig. 1B, right upper quadrant within left panel). Similarly, UVC-irradiated primary murine thymocytes showed a time-dependent rise in MDA-epitopes correlating with late apoptosis (Supplemental Fig. S1E).

**Supplemental Figure 2:**
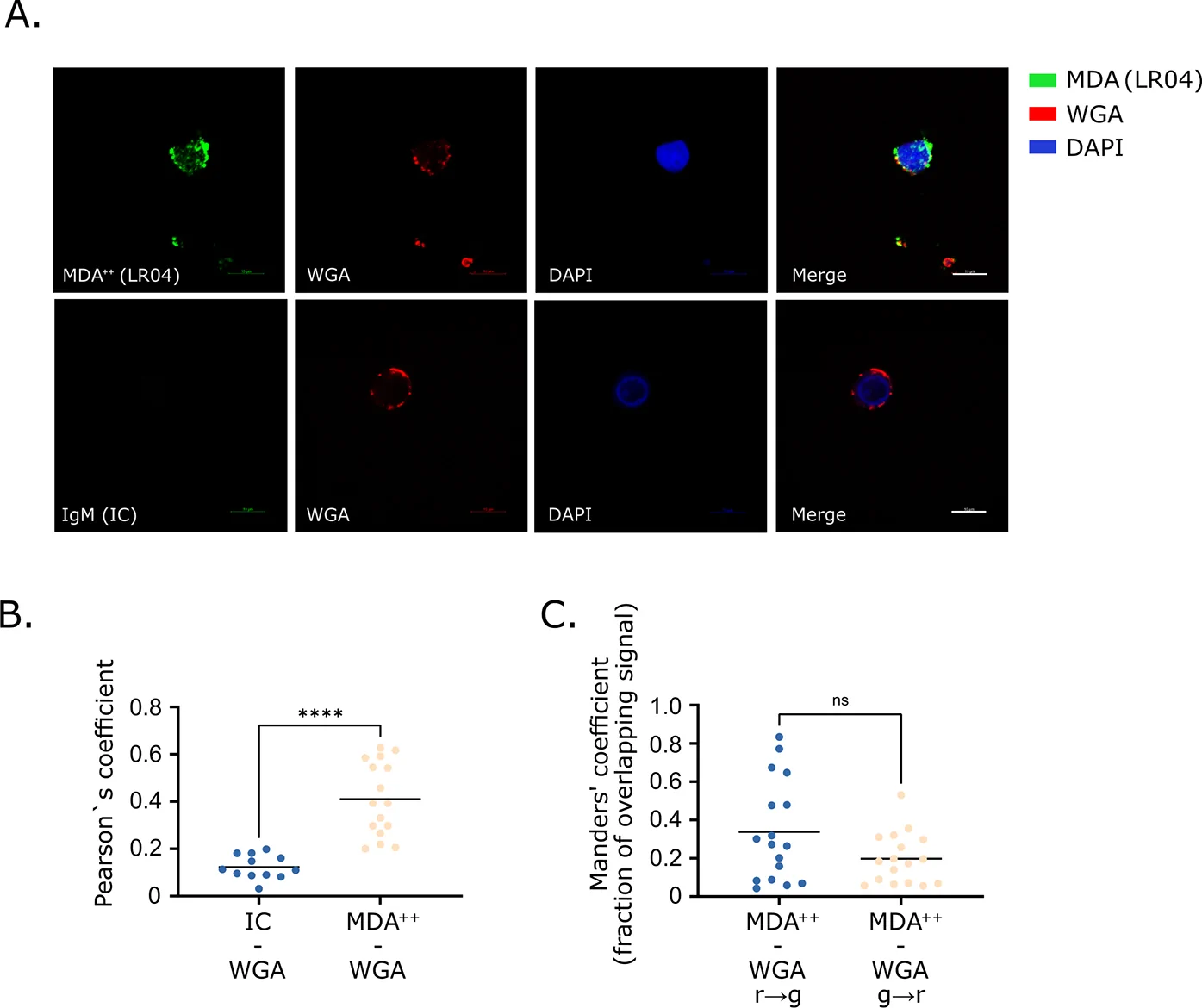


To characterize the surface appearance of MDA-epitopes on apoptotic cells we performed confocal microscopy of non-permeabilized UVC-irradiated Jurkat T cells stained with the membrane marker wheat-germ agglutinin (WGA, red signal), and the MDA-specific antibody LR04 or an isotype control (IC, green signal). Colocalization analysis (Pearson’s and Mander’s coefficients) of non-permeabilized cells demonstrates that MDA-epitopes initially appear on and colocalize with the membrane marker (Fig. 1C–E). At later stages, with extensive membrane damage, epitope abundance and spread throughout the cell, colocalization is reduced (Supplemental Fig. S2A–C).

Thus, MDA-epitopes are primarily associated with apoptotic cells with impaired membrane integrity, and this is independent of cell type and the apoptosis pathways triggered.

**Supplemental Figure 3:**
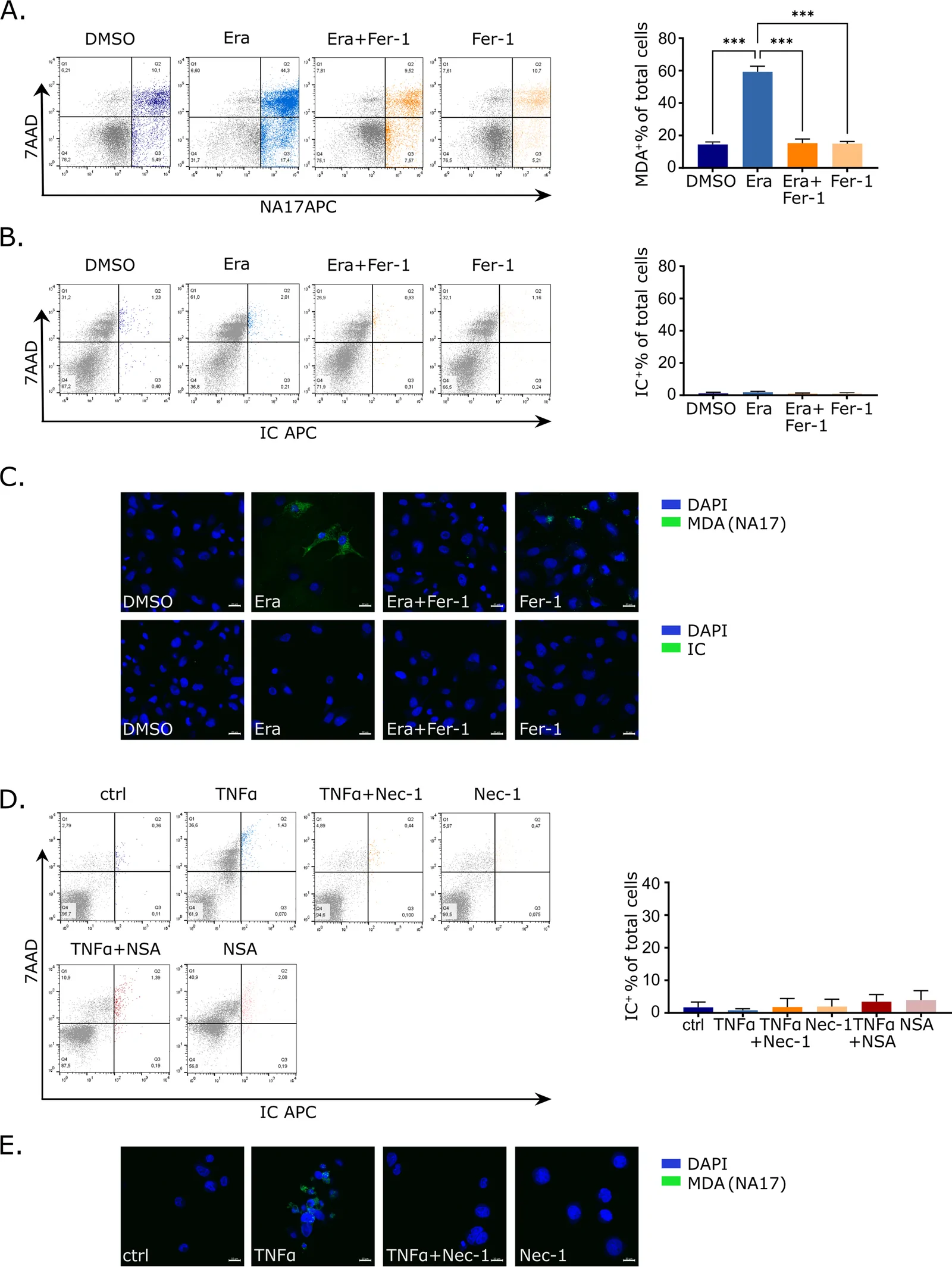


Given the strong association between membrane damage and MDA-epitope presence, we hypothesized that other PCD pathways involving membrane disruption would also result in the display of MDA-epitopes. We examined ferroptosis and necroptosis, both characterized by membrane damage and DAMP release (3, 36). Recently, one type of MDA-epitopes, a cyclic 4-methyl-1,4-dihy-dropyridine-3,5-dicarbaldehyde (MDHDC) structure, was demonstrated by immunocytochemistry using a mouse monoclonal IgG antibody, 1F83, on ferroptotic cells (37, 38, 39). To validate and expand these observations using our MDA-specific natural IgM antibodies, which detect a broader repertoire of MDA-epitopes, ferroptosis was induced in HT-1080 cells with erastin in the presence or absence of the ferroptosis inhibitor, ferrastatin-1 (Fer-1). Sixteen hours post-induction, flow cytometry confirmed MDA-epitope accumulation, with both IgM antibodies. These epitopes were detected on cells with damaged membrane (7AAD^+^), while concomitant Fer-1 treatment prevented this (Figs. 1F, Supplemental Fig. S3A, B). Fluorescence microscopy revealed a distinct thread-like pattern of MDA-epitopes in ferroptotic cells, absent in controls or isotype-stained cells (Figs. 1G, Supplemental Fig. S3C), supporting their presence during ferroptosis (38, 39).

To study the appearance of MDA-epitopes during necroptosis, Fas-associated death domain protein (FADD) deficient (FADD−/−) Jurkat T cells were stimulated with human TNFα in the presence or absence of inhibitors, necrostatin-1 (Nec-1) or necrosulfonamide (NSA). Necroptotic cells exhibited a marked MDA-epitope accumulation, particularly in conjunction with 7AAD-positivity (Fig. 1H), whereas inhibition by Nec-1 or NSA largely prevented it (Figs. 1H, Supplemental Fig. S3D). Furthermore, confocal microscopy of necroptotic FADD−/− Jurkat T cells demonstrated a punctate pattern of MDA-epitopes compatible with membrane disruption by pore-forming mixed lineage kinase domain-like (MLKL) protein oligomers - a necroptosis executor (Figs. 1I, Supplemental Fig. S3E).

Therefore, the appearance of MDA-epitopes coincides with cellular membrane deterioration independently of the initiated PCD signaling cascade.

### MDA-epitopes activate the classical complement pathway

Previously, we and others have shown that MDA-epitopes on apoptotic blebs and necrotic cells recruit regulators of the complement cascade, such as FH, complement factor H-related protein 1, 3 and 5 (FHR1, FHR3, and FHR5), but whether they directly activate complement cascades remains unclear (9, 11, 19, 20). To assess this, we monitored deposition of complement components from human serum on immobilized proteins modified with two commonly observed MDA-epitope forms, either simple MDA- or more immunodominant MAA-structures.

**Fig. 2:**
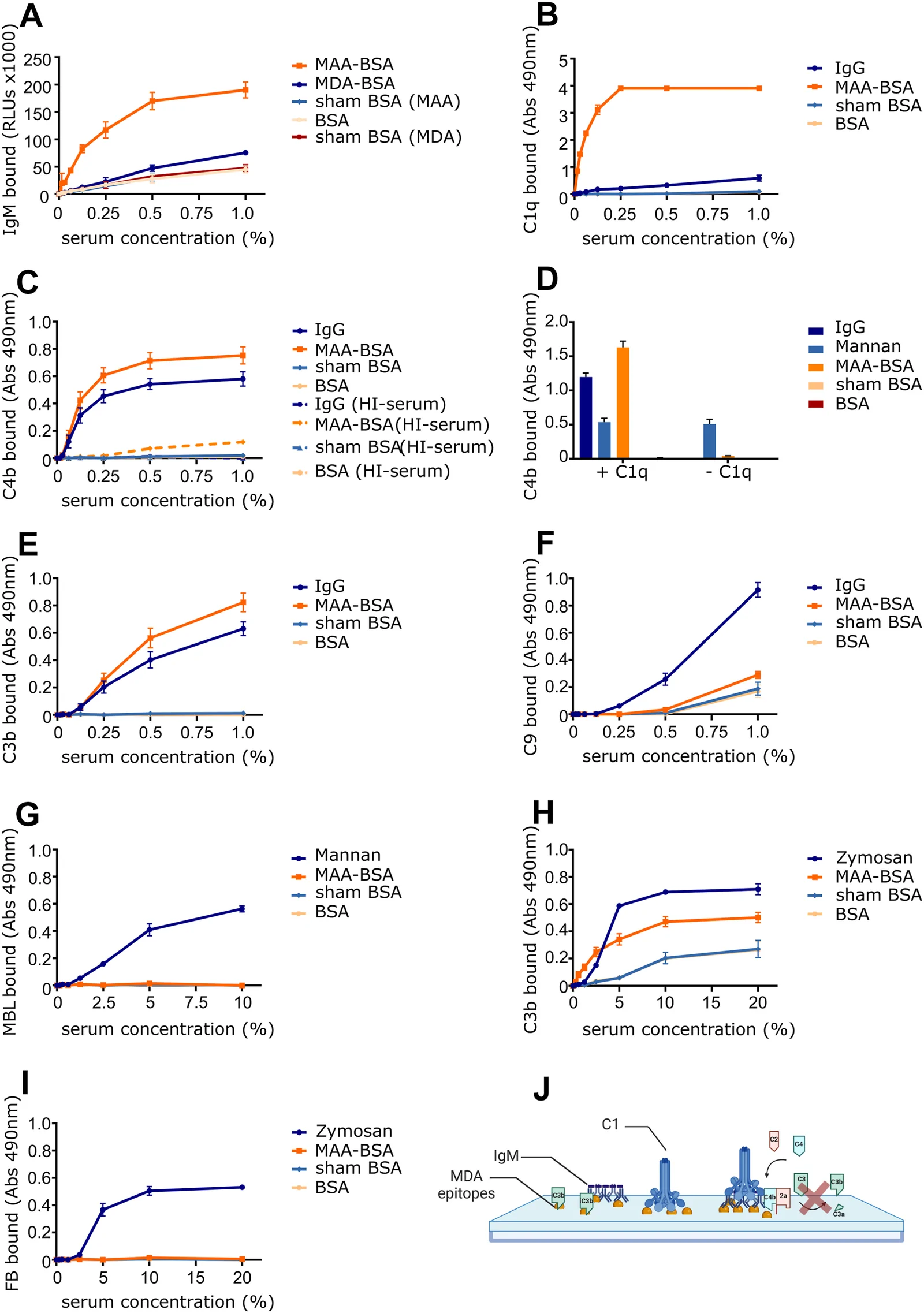
The MDA surfaces are recognized by complement components and activate the classical complement pathway. ELISA based assays for determining activation of all three complement pathways. Deposition of different complement components from increasing concentrations of normal (NHS) or heat-inactivated human serum (HI) to coated IgG, mannan, zymosan, MAA-BSA, MDA-BSA, sham BSA, or naïve BSA (BSA) was detected by specific antibodies. A–F: Activation of the classical complement pathway was demonstrated by deposition of (A) IgM, (B) C1q, (C) C4b from NHS and HI NHS, (D) C4b from C1q-depleted or restored NHS, (E) C3b, and (F) C9. Activation of the lectin pathway was determined by deposition of (G) mannan binding lectin (MBL). Activation of the alternative pathway was observed by deposition of (H) C3b, (I) Factor B (FB). Shown values are obtained from three independent experiments and expressed as mean ± SD. J: A schematic representation of the proposed consequences of the classical pathway activation on MDA-bearing surfaces.

**Supplemental Figure 4:**
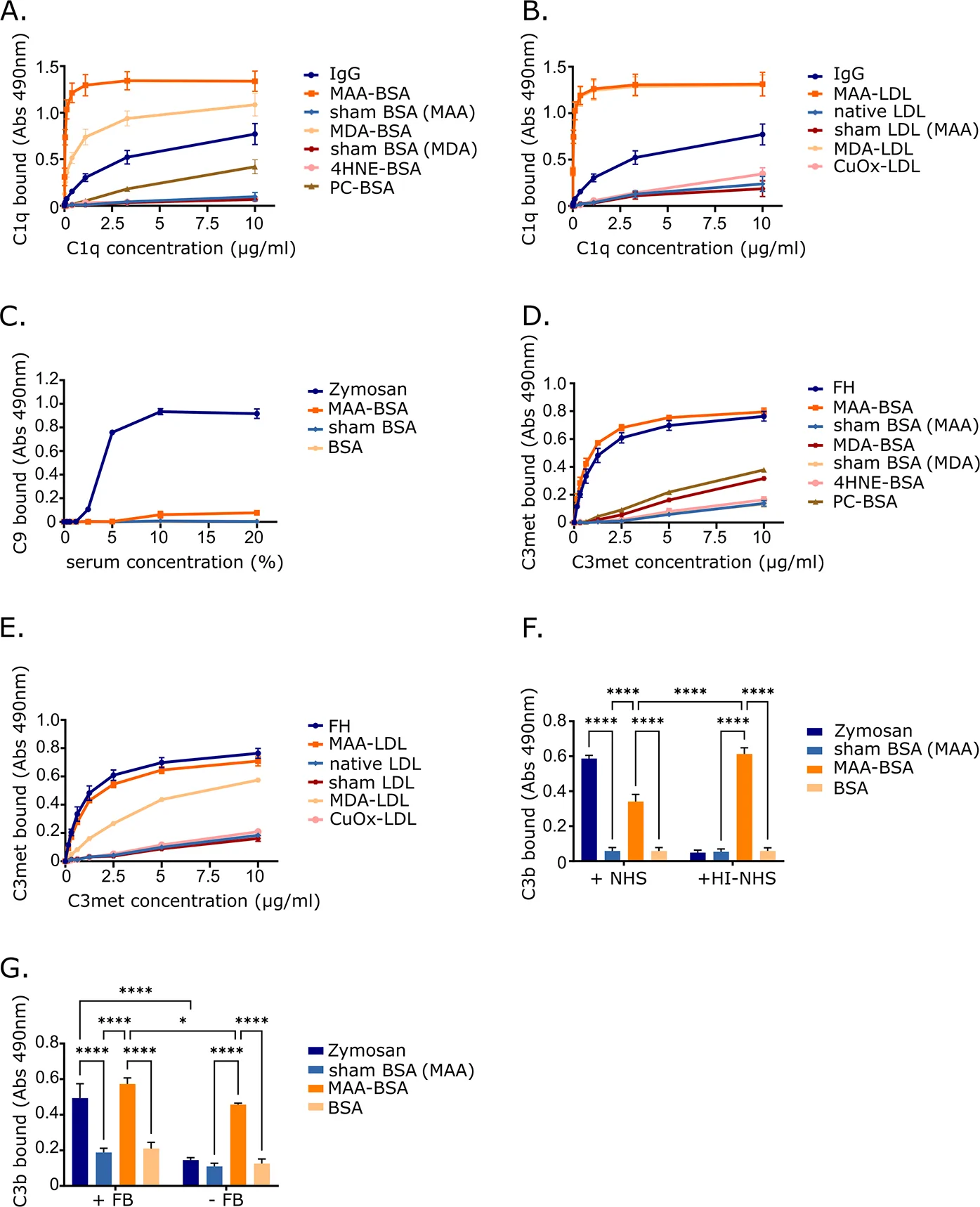


In contrast to unmodified BSA, MDA- and MAA-BSA mediated robust deposition of IgM from normal human serum (NHS) (Fig. 2A). Similarly, MAA-BSA showed strong C1q accumulation even at low serum concentrations, exceeding IgG controls, in contrast to unmodified or sham-modified BSA controls (Fig. 2B). Purified human C1q bound specifically to MDA- and MAA-modifications but not to other OSEs such as 4-hydroxynonenal (4-HNE) or phosphocholine (PC) (Supplemental Fig. S4A, B). This interaction was independent of the carrier protein, as both MDA- and MAA-modified BSA and LDL bound C1q, but dependent on adduct density (Supplemental Fig. S4A, B). Bovine serum albumin contains ∼60 lysine residues available for adduction with MDA- or MAA-haptens, whereas LDL, with ApoB alone harboring ∼360 lysines can undergo more extensive modification and possess a higher adduct density. Hence, differences in C1q binding between the less reactive MDA-adducts and the immunodominant MAA-adducts are more pronounced when BSA is used as a carrier (Supplemental Fig. S4A, B).

Potent binding of the C1 complex to MAA-carrying macromolecules resulted in classical pathway activation as demonstrated by the deposition of C4b from sera (Fig. 2C). No C4b deposition was observed when heat-inactivated (HI) serum with inactive complement (Fig. 2C) or serum depleted of C1q was used (Fig. 2D). In addition, MAA-modifications mediated deposition of C3b from serum (Fig. 2E) but not of C9 (Fig. 2F). MAA-BSA did not initiate the lectin pathway, at least not via MBL, which it did not bind (Fig. 2G). Furthermore, when we tested if MAA-adducts have the potential to activate alternative pathway, we found that MAA-BSA recruited C3b from serum (Fig. 2H) but did not yield deposition of Factor B (Fig. 2I) or C9 in contrast to zymosan, that was used as a positive control (Supplemental Fig. S4C). Moreover, purified C3met, which functionally corresponds to C3b, bound to MDA-epitopes, both MDA- and MAA-structures, independently of the protein carrier in a density dependent manner (Supplemental Fig. S4D, E). This observed C3b recruitment by MDA-epitopes was not dependent on complement activation since C3b was also deposited from HI-NHS or Factor B-deficient sera (Supplemental Fig. S4F, G). Interestingly, higher C3b deposition on MAA-carrying antigens in the presence of HI-NHS versus NHS suggests active inhibition in serum with functional complement (Supplemental Fig. S4F). Thus, MDA-epitopes bind C3b but do not activate the alternative pathway.

These findings clearly show that although recognized by the opsonins IgM, C1q and C3b, MDA-epitopes trigger only limited classical complement pathway activation up to the level of C4b or C3b cleavage (Fig. 2J).

### MDA-epitopes bind C4BP, the major inhibitor of the classical and lectin pathway

The limited classical pathway activation on MDA-epitopes suggested that they might recruit both activators (IgM, C1q, and C3b) but also C4BP, an inhibitor of the classical and lectin pathways.

**Fig. 3:**
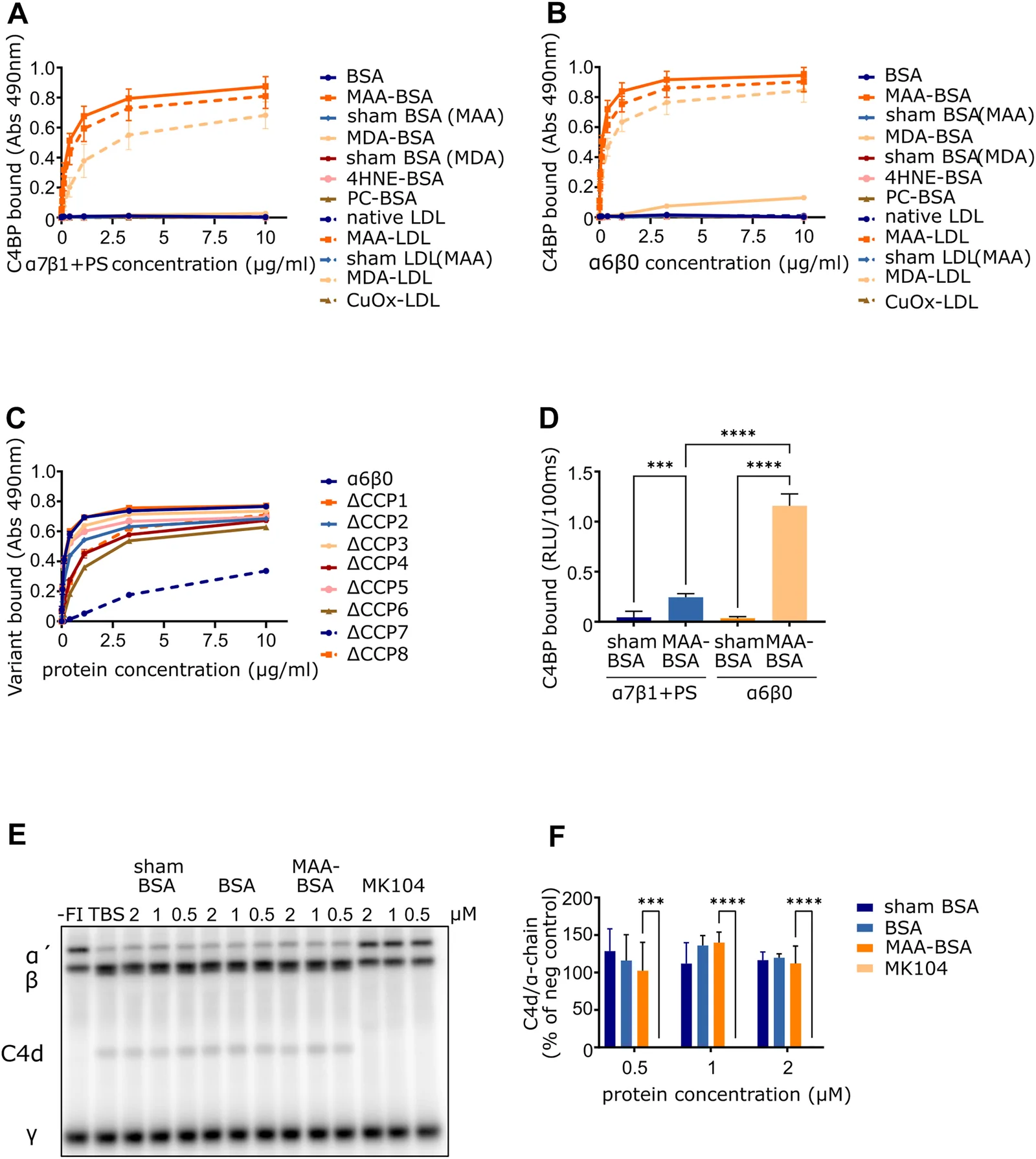
The MDA-epitopes recruit C4BP, a major inhibitor of the classical and lectin complement pathways. A: ELISA for binding of plasma purified α7β1 C4BP + PS in increasing concentrations to various OSE modifications of BSA and LDL, such as 4-hydroxynonenal BSA (4-HNE-BSA), MDA-BSA, MAA-BSA, phosphocholine-BSA (PC-BSA), MDA-LDL, MAA-LDL and CuOx-LDL (enriched in PC epitopes), N = 4. B: ELISA for binding of recombinant α6β0 C4BP in increasing concentrations to various OSE modifications of BSA and LDL, N = 4. C: ELISA for binding of α6β0 C4BP, and C4BP variants with deleted individual CCP domains (ΔCCP1, ΔCCP2, ΔCCP3, ΔCCP4, ΔCCP5, ΔCCP6, ΔCCP7 and ΔCCP8) to MAA-BSA, N = 3. D: ELISA for binding of C4BP variants (α7β1+PS and α6β0) at a concentration of 1.6 μM to MAA-BSA and sham-BSA, N = 3. E: C4b degradation assay in solution. ^125^I-labeled C4b was incubated with FI ± C4BP in the presence of increasing concentrations of BSA, MAA-BSA, or MK104 antibody. The amount of C4d fragments was determined by SDS-PAGE, N = three. F: Quantification of freshly generated C4d fragments bands represented as C4d/α-chain (percentage of negative control) ratio. Statistical differences were tested by two-way ANOVA, with Bonferroni’s multiple comparisons (∗∗∗*P*-value ≤ 0.001, ∗∗∗∗*P*-value ≤ 0.0001). Values are expressed as mean ± SD.

We tested binding of two major plasma C4BP isoforms: α7β1+ Protein S (PS) and acute phase α6β0 variant on MDA-epitopes and other OSEs. Both isoforms bound exclusively to MDA- and MAA-modified macromolecules, independent of carrier but dependent on epitope abundance (Figs 3A, B), indicating that C4BP α-chain mediates this interaction.

MDA-binding α-chains of C4BP consist of eight complement control protein (CCP) modules, among which the first four CCPs mediate their complement inhibitory function (40). Using recombinant single α-chain C4BP variants lacking individual CCP domains, we mapped the binding site to CCP7, as its removal markedly reduced binding to MAA-modifications (Fig. 3C). In addition, when added in equimolar amounts, the acute phase response variant of C4BP α6β0 bound more potently to MAA-modifications than α7β1+PS variant (Fig. 3D). Surface plasmon resonance assay confirmed the highest affinity of the C4BP α6β0 variant (K_D_ ∼ 2.2 ± 1.2 nM) to MDA-epitopes, several fold greater than α7β1, while the lowest affinity was observed for a single α-chain variant C4BPstopCCP8 with a stop codon inserted after CCP8, thus lacking the polymerization domain (Table 1). This observation suggests that the measured affinities of multimeric isoforms represent avidity rather than true affinity.

**Table 1:** The binding kinetics of different C4BP variants to MDA-epitopes

Protein	K_a_ (1/Ms) ×10^5^	Kd (1/s) ×10^−3^	KD (nM)
α7β1+PS	2.5 ± 3	1.3 ± 0.8	15.2 ± 2.1
α6β0	2.6 ± 0.6	0.5 ± 0.2	2.2 ± 1.2
C4BPStopCCP8	0.01 ± 0.01	0.2 ± 0.14	262 ± 157.2

**Supplemental Figure 5:**
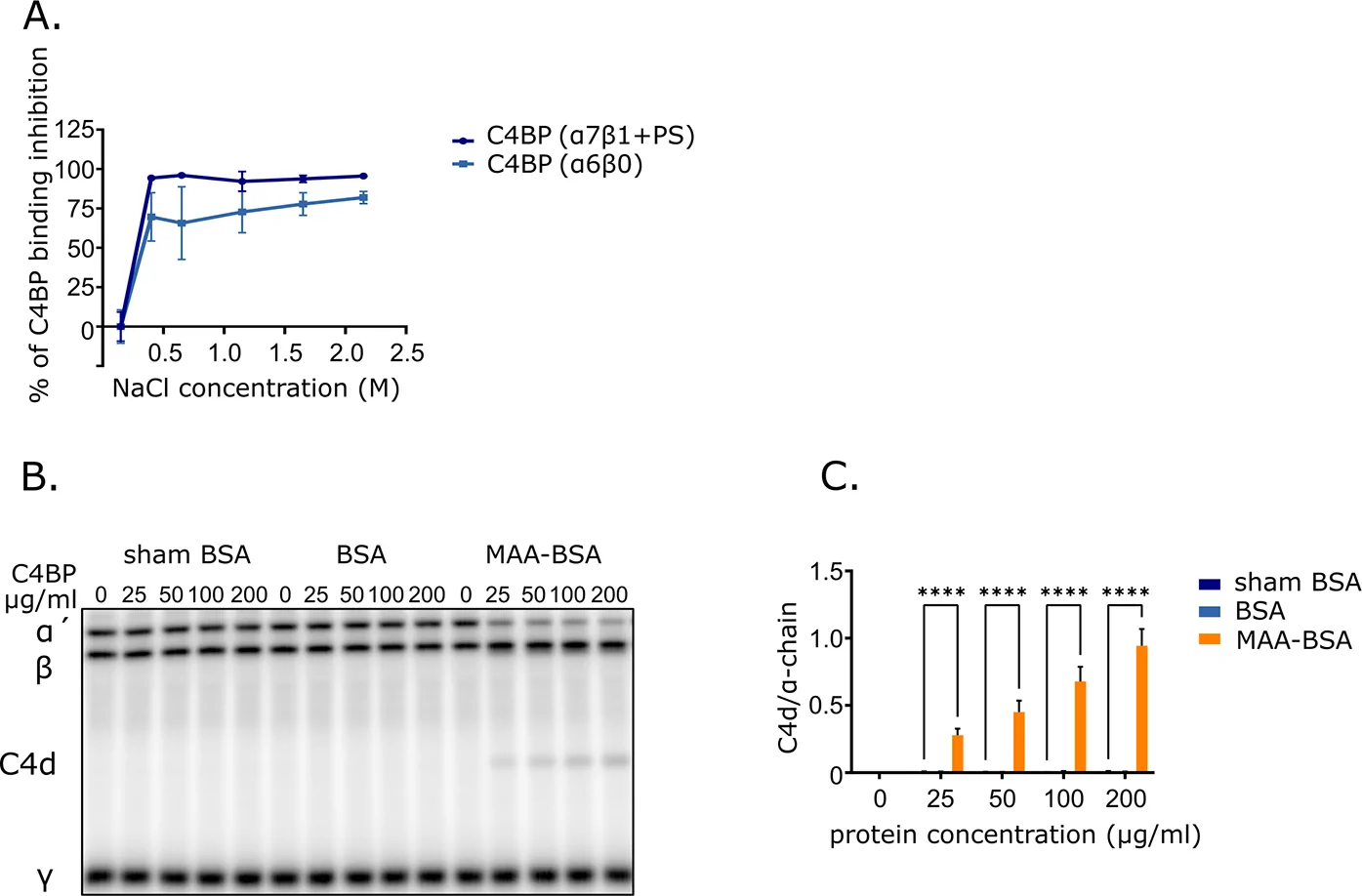


Binding assays under increasing NaCl concentrations demonstrated reduced binding of both C4BP variants (α7β1, α6β0) with MDA-epitopes indicating an interaction of ionic nature (Supplemental Fig. S5A).

The C4BP inhibits classical and lectin pathway activation by binding up to four molecules of C4b via CCPs 1–3 of four different α-chains, and acts as a cofactor for FI-mediated cleavage of C4b (84 kDa) into inactive C4d fragment (45 kDa) (40, 41). To test whether C4BP still retains this function when bound to MDA-epitopes, we performed a C4b degradation assay in solution incubating I^125^-labeled C4b, recombinant C4BP (α6β0), FI in the presence or absence of increasing concentrations of MAA-BSA, or BSA controls. Additionally, MK104, an antibody against CCP1 that blocks the binding of C4b to C4BP, served as a control (Fig. 3E). As expected, C4BP still promoted the formation of C4d even when bound to MAA-BSA as CCP7 mediates MDA-epitope binding while CCPs1–3 remain available for C4b interaction (Fig. 3E, F). Finally, plate-based assays confirmed that C4BP bound to MDA-epitopes inhibits classical pathway activation, whereas no C4d was generated on control BSA (Supplemental Fig. S5B, C).

Thus, MDA-epitopes bind C4BP variants via the CCP7 domain of the α-chain. Binding affinity is potentiated by C4BP’s multimeric structure and multiple MDA-binding sites, but reduced by the β-chain and Protein S. Once recruited to MDA-decorated surfaces, C4BP exerts its ability to act as an inhibitor of the classical and lectin pathways.

### MDA-epitopes mediate the recruitment of complement components on the surface of dying cells and affect their removal by phagocytes

**Fig. 4:**
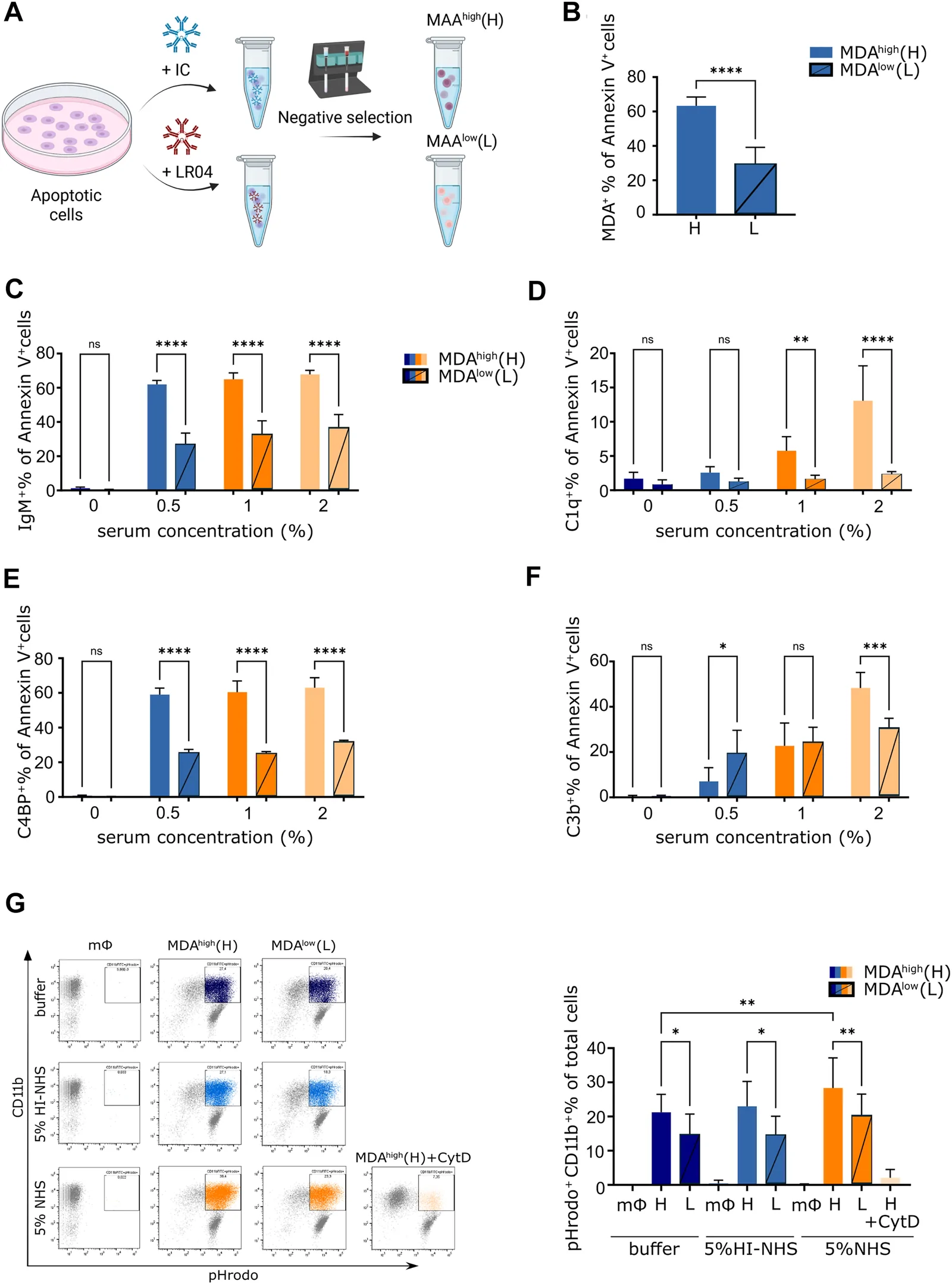
The presence of MDA-epitopes guides the deposition of complement components on dying cells and facilitates their clearance. A: Schematic representation of the immuno-depletion method developed to generate MDA^high^ (H) and MDA^low^ (L) cellular fractions. UVC-irradiated apoptotic cells were incubated with isotype control (IC) or LR04 (mouse mAb IgM against MDA-epitopes) and afterward with anti-IgM magnetic beads. When placed in a magnetic field, unbound apoptotic cells were separated from antibody-bound fractions using MS MACS columns (negative selection). B: Quantification of MDA-epitope presence between MDA^high^ (H) (no border on column, left) and MDA^low^ (L) (black border on column, right) cellular fractions by LR04 antibody in a flow cytometry-based assay (N = 8 independent experiments with duplicates). C–F.) Measuring opsonization by flow cytometry of MDA^high^ (H) (no border, left) and MDA^low^ (L) (bordered columns, right) fractions of dying cells by different opsonins detected from NHS (0.5%, 1%, or 2%) in a Ca^2+^containing buffer. C: Quantification of IgM deposition obtained by three independent experiments with biological duplicates. D: Quantification of C1q deposition, generated by three experiments with biological duplicates. E: Quantification of C4BP deposition. F) Quantification of C3b deposition, measured by four experiments with technical duplicates. Bars represent mean ± SD values. G) The flow cytometry-based assay to determine efferocytosis of cells with different amounts of MDA-epitopes in the absence and presence of 5% heat-inactivated (HI) or 5% normal human serum. pHrodo labeled MDA^high^ (H) and MDA^low^ (L) fractions of apoptotic Jurkat T cells were incubated with PMA-matured THP1 macrophages (CD11b^+^) (1:1 ratio) for 1 h (left panel). Quantification of efferocytosis assay (right panel) from four independent experiments using triplicates. Bars represent mean ± SD values. Statistical differences were tested by one or two-way ANOVA, with Tukey’s multiple comparisons (∗*P*-value ≤ 0.05, ∗∗*P*-value ≤ 0.01, ∗∗∗*P*-value ≤ 0.001, ∗∗∗∗*P*-value ≤ 0.0001, ns = not significant).

**Supplemental Figure 6:**
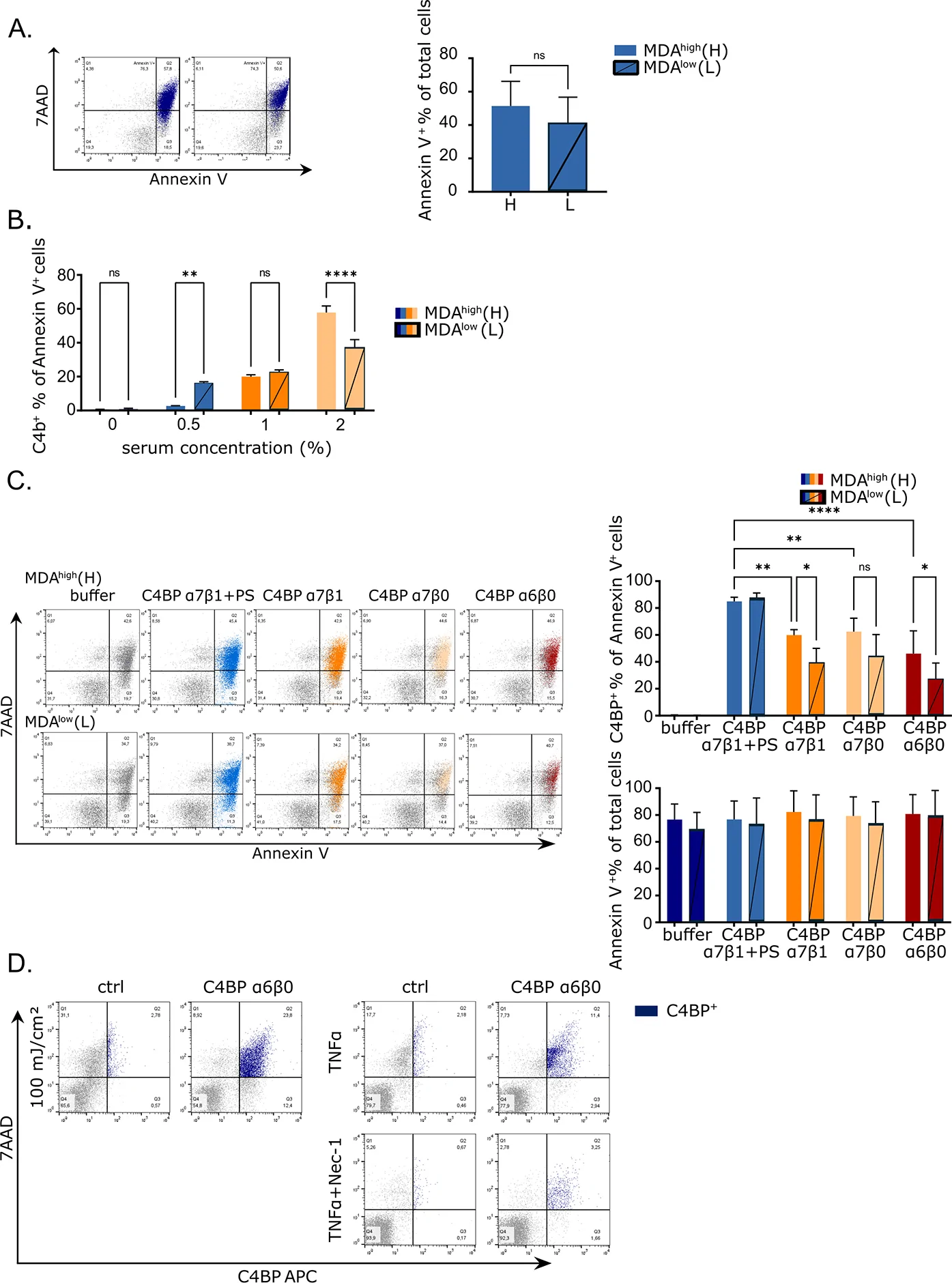


We next wanted to evaluate if MDA-epitopes present on dying cells serve as hubs for the recruitment of IgM, C1q, C3b, C4b, and C4BP, thereby modulating opsonization. To test this, we incubated apoptotic Jurkat T cells with an MDA-specific antibody (LR04) or isotype control coupled to magnetic beads to immunodeplete MDA-epitope^+^ apoptotic cells. After negative selection on magnetic columns, we collected two fractions of apoptotic Jurkat T cells, defined by having high (MDA^high^ (H), IC-unbound) or low (MDA^low^ (L), LR04-unbound) levels of MDA-epitopes (Fig. 4A, B), both having a similar extent of apoptosis as measured by exposure of PtdSer (Supplemental Fig. S6A). Incubation of both fractions with NHS revealed significantly lower deposition of IgM (Fig. 4C), C1q (Fig. 4D), C4BP (Fig. 4E), C3c (Fig. 4F) and C4c (Supplemental Fig. S6B) on MDA^low^ (L) cells, suggesting that MDA-epitopes on apoptotic cells are important tags and required for opsonization.

Protein S bound to the β-chain of plasma C4BP (α7β1+PS) anchors this isoform to apoptotic cells via Phosphatidylserine (PtSer) in a Ca^2+^ dependent manner (42). To investigate whether MDA-epitopes also act as a docking site for C4BP on apoptotic cell surfaces, plasma-purified (α7β1+PS) and recombinant variants lacking PS (α7β1, α7β0, α6β0) of C4BP were incubated with apoptotic Jurkat T cells in a Ca^2+^-containing buffer. In the presence of Ca^2+^, apoptotic Jurkat T cells enriched in MDA-epitopes (MDA^high^ (H)) bound to all C4BP variants (Supplemental Fig. S6C), whereas MDA^low^ (L) apoptotic cells showed significantly reduced recruitment of two variants lacking a β-chain or PS (α7β0, α6β0). No MDA-dependent recruitment of the α7β1+PS C4BP variant to apoptotic cells was observed, probably due to its ability to be recruited to PtSer via PS. The extent of apoptosis and availability of PtSer were similar in all used fractions (Supplemental Fig. S6C, right panel, lower graph). Apoptotic and necroptotic FADD−/− Jurkat T cells bound the α6β0 variant of C4BP in a calcium-independent manner (Supplemental Fig. S6D), suggesting that under acute phase conditions with higher tissue damage, MDA-epitopes may serve as hubs for the attachment of the newly synthesized α6β0 C4BP isoform. To study if the differences in complement proteins and IgM deposited on MDA^high^ (H) and MDA^low^ (L) apoptotic cells would affect their uptake by phagocytes, we performed in vitro efferocytosis assays. Phorbol-12-myristat-13-acetat (PMA) matured THP1 like macrophages were fed pHrodo stained MDA^low^ (L) and MDA^high^ (H) fractions of apoptotic Jurkat T cells in the absence and presence of 5% HI-NHS or NHS. The engulfment by CD11b^+^ THP1 like macrophages was quantified by pHrodo fluorescence using flow cytometry. Across all conditions, THP1 like macrophages preferentially internalized apoptotic cells with a higher amount of MDA-epitopes (MDA^high^ (H)) compared to MDA^low^ (L) ones (Fig. 4G). However, the strongest uptake of MDA^high^ (H) cells was observed only when serum with fully functional complement was used. As a control, engulfment of MDA^high^ (H) cells was completely inhibited when Cytochalasin was used, confirming actin-dependent phagocytosis (Fig. 4G).

Our data demonstrate that MDA-epitopes are additional "eat-me" signals that modulate opsonization and promote clearance of dying cells with lost membrane integrity.

### Clearance rate of dying cells by primary macrophages is reliant on MDA-epitopes and is modulated by the recruitment of C4BP

**Fig. 5:**
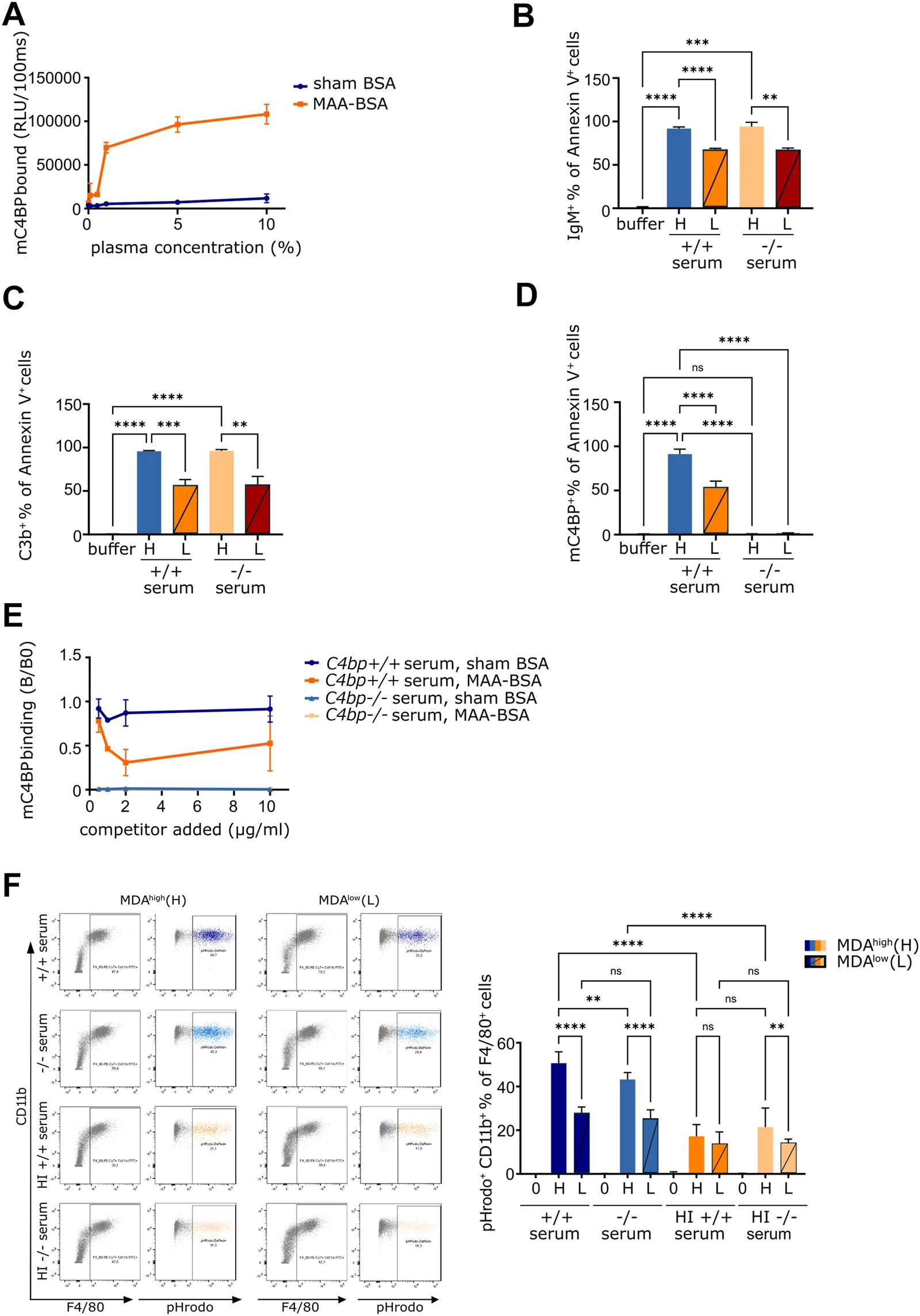
The MDA-epitopes on dying cells are recognized by murine complement system, which orchestrates their removal by phagocytes. A: ELISA for binding of murine C4BP (mC4BP) from plasma to MAA-BSA and not to sham BSA. Values are obtained from two independent experiments, each having duplicate values and expressed as mean ± SD. B–D: Measuring opsonization by flow cytometry of MDA^high^ (H) (no border, left) and MDA^low^ (L) (bordered columns, right) fractions of dying cells by different opsonins detected from 5% normal mouse serum (NMS) in a Ca^2+^ containing buffer. B: Quantification of IgM deposition (four independent experiments, each with duplicate values). C: Quantification of C3b deposition (three independent experiments, each with duplicate values). D: Quantification of C4BP deposition (three independent experiments, each with duplicate values). E: The flow cytometry-based assay demonstrates competition in the binding of mC4BP to apoptotic cells in the presence of increasing concentrations of MAA-BSA. The obtained data from four independent experiments are calculated as the ratio of the percentage of the mC4BP^+^ cells for a particular concentration of competitor (B) and the basal binding, where no competitor was added (B0). F: The flow cytometry-based assay to determine efferocytosis of cells with different amounts of MDA-epitopes in the *C4bp+/+* or *C4bp−/−* serum. Cell culture media (0) or pHrodo-labeled MDA^high^ (H) and MDA^low^ (L) fractions of apoptotic RAW cells were incubated with thioglycolate-elicited macrophages (0.8:1 ratio) for 1 h in the absence or presence of 5% normal or heat-inactivated (HI) *C4bp+/+* or C4bp*−/−* serum. In the left panel two gates are presented: the parental one demonstrating CD11b^+^ and F4/80^+^ of engulfing cells, and the following one showing efferocytosis (pHrodo^+^) within the parental one. In the right panel corresponding quantifications are shown. Bars represent mean ± SD values of triplicate determinations pooled from four independent experiments. Statistical differences were tested by one-way ANOVA, with Tukey’s multiple comparisons (∗∗*P*-value ≤ 0.01, ∗∗∗*P*-value ≤ 0.001; ∗∗∗∗*P*-value ≤ 0.0001, ns = not significant).

**Supplemental Figure 7:**
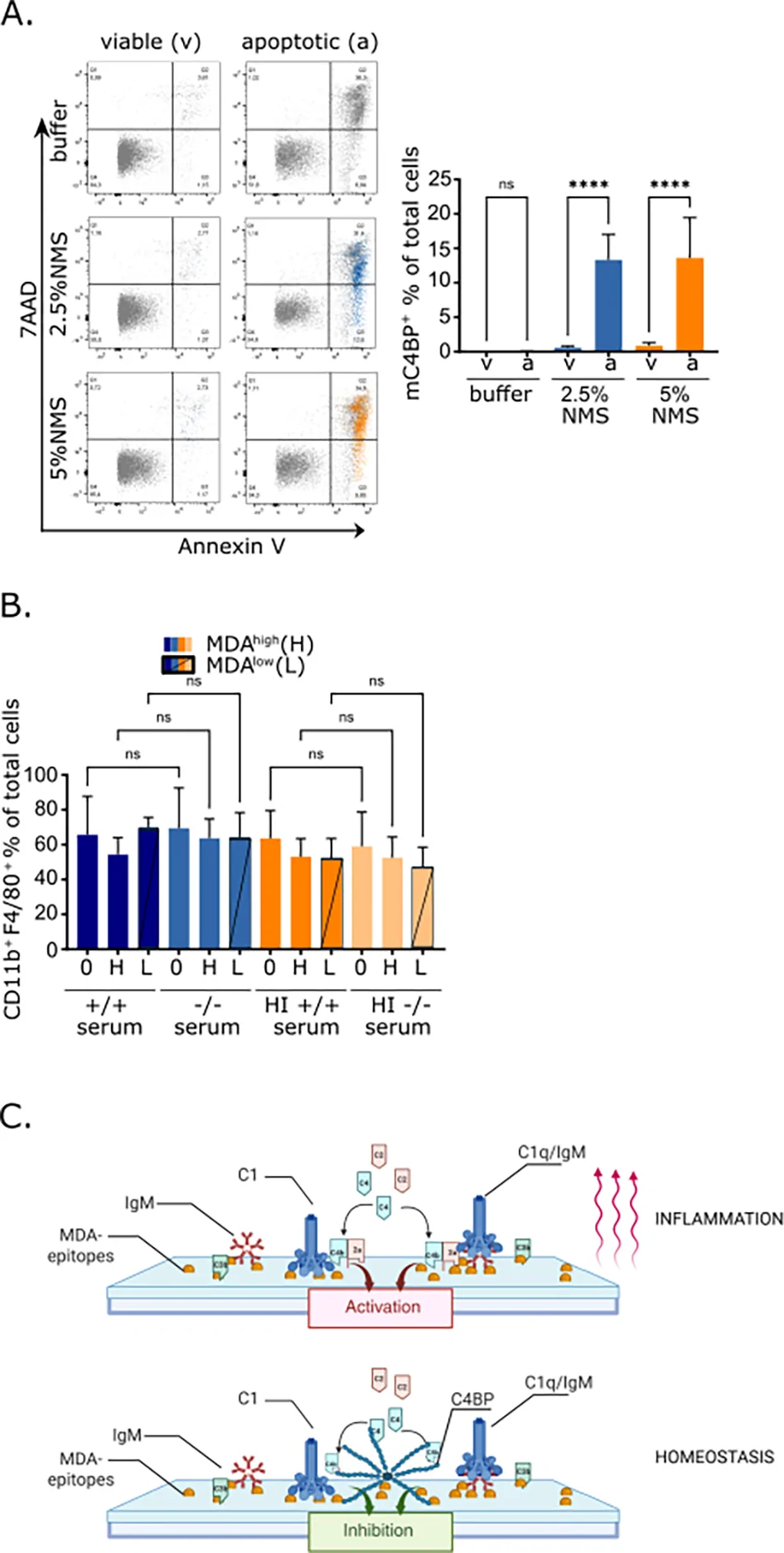


To study the effect of interaction between C4BP and MDA-epitopes as well as the uptake of dying cells by primary phagocytes in a more physiologically relevant setup, we performed experiments using serum of wild type and C4BP-deficient mice. Murine C4BP (mC4BP) shows some structural differences compared to the human counterpart. It consists only of seven α-chains, each containing six CCPs, lacking orthologues of the human CCPs 5 & 6 but possesses CCP7 which binds MDA-epitopes (43, 44, 45). Using ELISA, we confirmed that plasma-derived mC4BP was deposited on MAA-BSA in a concentration-dependent manner, but not on sham BSA (Fig. 5A). Next, the presence of MDA-epitopes on dying cells also modulated opsonin deposition such as murine IgM and C3c. After 30 min of incubation of MDA^high^ (H) and MDA^low^ (L) fractions of apoptotic murine RAW 264.7 cells with 5% murine *C4bp+/+* and *C4bp−/−* serum, we observed higher deposition of IgM (Fig. 5B) and C3c (Fig. 5C) on MDA^high^ (H) cells, and this effect was not affected by mC4BP. Similarly to human β-chain lacking C4BP variants, a higher amount of mC4BP from the plasma was deposited on MDA^high^ (H) apoptotic cell surfaces (Fig. 5D). Moreover, mC4BP bound to both apoptotic RAW264.7 cells (mouse cells, Fig. 5D) and Jurkat T cells (human cells, Supplemental Fig. S7A), primarily on Annexin V^+^, 7AAD^+^ populations enriched in MDA-epitopes (Supplemental Fig. S7A, left panel,). Finally, a flow cytometry-based competition assay confirmed MDA-epitopes as complement recruitment hubs, where binding of serum mC4BP to apoptotic RAW264.7 cells was diminished by increasing concentrations of MAA-BSA, but not sham BSA (Fig. 5E). Finally, to assess the contribution of C4BP recruitment by MDA-epitopes and consequences of it in efferocytosis, we incubated thioglycolate-elicited murine macrophages with pHrodo stained MDA^high^ (H) and MDA^low^ (L) fractions of apoptotic RAW264.7 cells in the presence or absence of normal and HI *C4bp+/+* and *C4bp−/−* mouse serum (NMS). In the presence of normal serum of *C4bp+/+* and *C4bp−/−* mice, macrophages engulfed more of MDA^high^ (H) cells compared to MDA^low^ (L) cells. Moreover, the presence of C4BP enhanced the clearance of (MDA^high^ (H)) cells but had no effect on MDA^low^(L) cell removal (Fig. 5F). This effect was lost when heat-inactivated serum was used (Fig. 5F). No differences in the percentage of CD11b^+^F4/80^+^ macrophages between different conditions were observed (Figs. 5F and Supplemental Fig. S7B). The higher apoptotic cell clearance in the murine system likely reflects intrinsic differences between the macrophage models. Thioglycolate-elicited peritoneal macrophages have prior exposure to apoptotic cells, unlike PMA-differentiated THP-1 macrophage-like cells. As a result, their efferocytic capacities differ — ∼50% versus ∼25%.

In this more physiologically relevant murine setup, our data show that mC4BP binding to MDA-decorated dying cells augments their removal by a mechanism that involves controlled activation of the classical complement pathway.

## Discussion

The presence of the MDA-epitopes on damaged structures, including apoptotic cells, a subset of extracellular vesicles, and oxidized lipoproteins, in acute or chronic pathologies has been repeatedly demonstrated (4, 9, 10, 12, 14). These epitopes represent a class of DAMPs, and we have previously found that several cellular and humoral components of innate immunity have the capacity to recognize, bind, and neutralize their pro-inflammatory effects. The impairment of these processes, either due to genetic deficiency, mutation, or simply exhaustion, has been linked to the development of chronic diseases (9, 19, 24, 25, 46). We now demonstrate that MDA-epitopes represent additional “eat me” signals on dead cells since major PCD pathways, including apoptosis, ferroptosis, and necroptosis in various primary cells and cell lines lead to their surface exposure, resulting in their recognition by humoral immune components, most prominently IgM and complement. Following recognition, these factors coordinate the “limited” activation of the classical complement pathway on damaged surfaces, resulting in MDA-mediated opsonization and engulfment by phagocytic cells demonstrating their physiological significance (Supplemental Fig. S7C).

In multicellular organisms, programmed cell death and efficient clearance of cellular debris are a necessity for survival (1, 2). However, different PCD pathways induce distinctive changes in cellular membranes affecting surrounding cells and phagocytic response (36, 47). When clearance fails, persistence and accumulation of dying cells that have lost their membrane integrity can result in more robust inflammatory signatures and dysregulated immune responses (6, 47, 48). Although the presence of MDA-epitopes on apoptotic or necrotic cells has been shown (4, 9, 10, 14, 19), here we demonstrate that late apoptotic cells with damaged membranes are major carriers of these epitopes. Generally, these cells with leaky membranes are particularly hazardous because they are considered to be a major source of intracellular autoantigens (49). Next to apoptosis, we also analyzed the presence of MDA-epitopes on ferroptotic and necroptotic cells. Ferroptosis is an iron-dependent PCD where the accumulation of reactive oxygen species leads to extensive lipid peroxidation, driving cellular death, while necroptosis is initiated by activation of receptor-interacting protein RIP1/RIP3 complexes that trigger translocation of MLKL into the cellular membrane, leading to lysis (3, 36, 50, 51). Using two different well-characterized MDA-specific natural IgM antibodies, which can detect a broader repertoire of MDA-epitopes, we could document by flow cytometry and immunocytochemistry that cells undergoing any of these lytic PCDs were enriched in MDA-epitopes, suggesting that MDA-epitopes might be warning tags that instruct phagocytes about potentially dangerous and highly inflammatory DAMP content (10, 24, 26, 28). Recently, the presence of MDA-epitopes was demonstrated in ferroptotic cells by similar methods, consistent with our findings (23, 24, 25). For the future, it would be of interest to understand the intracellular mechanisms leading to the initial generation of MDA-epitopes as well as their exact origin and location on membranes.

In general, apoptotic cells are swiftly scavenged by surrounding phagocytes after their PtdSer exposure on the cellular membrane. In contrast, the clearance of secondary necrotic cells with a leaky membrane (52) is less understood. Considering the ubiquitous occurrence of MDA-epitopes, our data suggests that these structures are important mediators of dying cell removal. In line with this, plasma levels of IgM antibodies against MDA-epitopes have been inversely associated with atherosclerotic cardiovascular disease and proposed as markers for risk prediction and discrimination, alongside traditional risk factors. MDA-specific IgM antibodies are thought to neutralize pro-inflammatory effects of oxidized lipoproteins and to support vascular homeostasis by promoting complement-mediated opsonization and non-inflammatory clearance of damaged cells (53, 54, 55, 56). In support of the latter, low MDA-specific IgM levels were reported to be associated with higher necrotic core volumes in coronary arteries of CHD patients (57). Indeed, we have previously shown that an MDA-specific IgM antibody has the capacity to enhance uptake of apoptotic cells in mice in vivo (26), and that atherosclerosis-prone mice with genetically high levels of OSE-specific IgM antibodies have less atherosclerosis with smaller necrotic areas consistent with more efficient efferocytosis (58). More recently we have shown the capacity of MDA-specific natural IgM to limit the procoagulant and NETogenic potential of MDA^+^ extracellular vesicles, although the contribution of complement in these effects remain unknown (10, 28, 59). Therefore, we focused on understanding their recognition by humoral immunity in more depth. In addition to natural IgM, we and others have previously identified C3a, FH, FHR5, FHR3, and FHR1 as MDA-epitope binders, but did not assess the role of complement in the recognition and clearance of dying cells. We now found that MDA-epitopes initiate the classical pathway cascade by binding to C1q and IgM and — importantly — that this activation is halted before the propagation phase (alternative pathway activation) maybe due to efficient recruitment of C4BP, especially the isoform lacking the β-chain (α6β0), which is induced in settings of acute inflammation (60). This is possible as C4BP binding to MDA-epitopes is mediated via CCP7 located on the α-chain of C4BP. Once bound to MDA-epitopes, either in solution or on surfaces, C4BP can act as a cofactor to FI in C4b degradation and inhibit downstream complement activation. Furthermore, although bound by many components of the alternative pathway (C3a, C3b, FH, FHRs 1, 3 & 5), MDA-epitopes did not demonstrate activation of this pathway in our experimental settings (9, 11, 19, 20). Thus, our findings provide mechanistic insights into how MDA-specific IgM can mediate anti-inflammatory and atheroprotective effects despite the potent complement activation capacity of IgM.

Notably, we demonstrate that the repertoire of opsonins deposited on late apoptotic cells with loss of membrane integrity, partially depends on the presence of MDA-epitopes, as we observed different amounts of IgM, C1q, C4BP, C3b, and C4b on MDA^high^ (H) versus MDA^low^ (L) dying cells. Enhanced deposition of C3b and C4b was noted at lower serum concentrations on MDA^low^ (L) cells, which had fewer IgM or C1q, suggesting that in that case the lectin pathway might be a major driver of their deposition, since pattern recognition receptors of the lectin pathway can bind other DAMPs, such as DNA (61, 62, 63, 64).

Differential opsonization of dying cells by complement proteins affects their recognition and engulfment by macrophages as well as mechanisms of cargo processing and the macrophage responses afterwards (65, 66, 67, 68, 69). MDA-epitopes can be recognized by scavenger receptors, and we now show that elevated levels of MDA-epitopes on dying cells, together with complement opsonins recognizing them, distinctly “flavor” dying cells surfaces and thereby substantially enhance their removal by phagocytic cells in both human and murine assays (2, 15). Moreover, this effect is further aided by the presence of C4BP, composed solely of α-chains (murine C4BP and acute variant of human C4BP). In contrast, human C4BP variants with β-chain (α7β1 ± PS) have been shown to slow down efferocytosis by blocking phosphatidylserine recognition (70). These opposing functions between the two major C4BP isoforms is further supported by findings showing that human monocyte-derived dendritic cells exposed to β-chain–deficient C4BP variants retain high endocytic activity while adopting an anti-inflammatory phenotype (71, 72). Therefore, the local ratio C4BP variants may, in a cell- and context-dependent manner, modulate inflammation. Our data additionally demonstrate that the pro-efferocytotic effect of mC4BP is independent of CCP6-mediated immunomodulation, as this domain is absent in murine C4BP (72). Furthermore, C4BP opsonization influences cargo degradation and trafficking, as shown for amyloid fibrils, suggesting analogous effects might affect apoptotic cells bearing different amounts of MDA-epitopes (66, 73). Taken together, our findings deepen the understanding of the multifaceted role of C4BP in maintaining homeostasis and facilitating efficient waste removal.

Finally, we show that dying cells decorated with MDA-epitopes to a different extent and bound by different amounts of associated opsonins undergo distinctive uptake by macrophages, further supporting the observation that local availability of complement components at sites of sterile inflammation matters (74). However, several limitations should be acknowledged. Although complement-dependent clearance of MDA epitope–decorated cells was consistently observed in both human and murine systems, the present study was conducted entirely in vitro, using a human cell line and murine primary macrophages as representative phagocytes. Consequently, the contribution of MDA-recognition by humoral immunity in efferocytosis in vivo in different pathophysiological settings remains to be established.

Our data define MDA-epitopes as “eat me” signals of different types of dying cells with lost membrane integrity and thereby as an important mediator of waste removal and homeostasis. Importantly, we demonstrate the capacity of MDA-adducts to trigger complement activation and the important role and hierarchy of specific complement components in mediating this effect. The recruitment of specific opsonins and complement components, especially the complement regulator C4BP, by MDA-epitopes represents a key mechanism for an orchestrated sensing of OSE to mediate homeostatic housekeeping functions without excessive pro-inflammatory complement activation. Impaired or exhausted complement-dependent clearance of these dying cells, may result in adverse proinflammatory effects mediated by the same OSEs via pattern recognition receptors.

## Ethics

All experimental procedures were approved by the Animal Ethics Committee of Austrian Ministry of Science (BMWFM-66.009/0188-WF/V/3b/2015) or by the Ethics Committee of the Medical University of Vienna (EK1845/2015) and conducted following the guidelines for Good Scientific Practice established by the Medical University of Vienna.

## Data availability

The data, reagents and further methodological details are available from the corresponding author on reasonable request and with approval from JLR. Correspondence and requests for materials should be addressed to christoph.binder@meduniwien.ac.at.

## Supplemental data

This article contains supplemental data.

## Conflict of interest

The authors declare that they have no conflicts of interest with the contents of this article.

## References

[1] Doran A.C., Yurdagul A., Tabas I. (2020). Efferocytosis in health and disease. Nat. Rev. Immunol..

[2] Arandjelovic S., Ravichandran K.S. (2015). Phagocytosis of apoptotic cells in homeostasis. Nat. Immunol..

[3] Bedoui S., Herold M.J., Strasser A. (2020). Emerging connectivity of programmed cell death pathways and its physiological implications. Nat. Rev. Mol. Cell Biol..

[4] Chang M.K., Binder C.J., Miller Y.I., Subbanagounder G., Silverman G.J., Berliner J.A. (2004). Apoptotic cells with oxidation-specific epitopes are immunogenic and proinflammatory. J. Exp. Med..

[5] Elliott M.R., Chekeni F.B., Trampont P.C., Lazarowski E.R., Kadl A., Walk S.F. (2009). Nucleotides released by apoptotic cells act as a find-me signal to promote phagocytic clearance. Nature.

[6] Fadok V.A., Voelker D.R., Campbell P.A., Cohen J.J., Bratton D.L., Henson P.M. (1992). Exposure of phosphatidylserine on the surface of apoptotic lymphocytes triggers specific recognition and removal by macrophages. J. Immunol..

[7] Mueller R.B., Sheriff A., Gaipl U.S., Wesselborg S., Lauber K. (2007). Attraction of phagocytes by apoptotic cells is mediated by lysophosphatidylcholine. Autoimmunity.

[8] Truman L.A., Ford C.A., Pasikowska M., Pound J.D., Wilkinson S.J., Dumitriu I.E. (2008). CX3CL1/fractalkine is released from apoptotic lymphocytes to stimulate macrophage chemotaxis. Blood.

[9] Alic L., Papac-Milicevic N., Czamara D., Rudnick R.B., Ozsvar-Kozma M., Hartmann A. (2020). A genome-wide association study identifies key modulators of complement factor H binding to malondialdehyde-epitopes. Proc. Natl. Acad. Sci. U. S. A..

[10] Amir S., Hartvigsen K., Gonen A., Leibundgut G., Que X., Jensen-Jarolim E. (2012). Peptide mimotopes of malondialdehyde epitopes for clinical applications in cardiovascular disease. J. Lipid Res..

[11] Irmscher S., Brix S.R., Zipfel S.L.H., Halder L.D., Mutluturk S., Wulf S. (2019). Serum FHR1 binding to necrotic-type cells activates monocytic inflammasome and marks necrotic sites in vasculopathies. Nat. Commun..

[12] Miller Y.I., Choi S.H., Wiesner P., Fang L., Harkewicz R., Hartvigsen K. (2011). Oxidation-specific epitopes are danger-associated molecular patterns recognized by pattern recognition receptors of innate immunity. Circ. Res..

[13] Horkko S., Miller E., Dudl E., Reaven P., Curtiss L.K., Zvaifler N.J. (1996). Antiphospholipid antibodies are directed against epitopes of oxidized phospholipids. Recognition of cardiolipin by monoclonal antibodies to epitopes of oxidized low density lipoprotein. J. Clin. Invest..

[14] Tsiantoulas D., Perkmann T., Afonyushkin T., Mangold A., Prohaska T.A., Papac-Milicevic N. (2015). Circulating microparticles carry oxidation-specific epitopes and are recognized by natural IgM antibodies. J. Lipid Res..

[15] Binder C.J., Papac-Milicevic N., Witztum J.L. (2016). Innate sensing of oxidation-specific epitopes in health and disease. Nat. Rev. Immunol..

[16] Duryee M.J., Klassen L.W., Schaffert C.S., Tuma D.J., Hunter C.D., Garvin R.P. (2010). Malondialdehyde-acetaldehyde adduct is the dominant epitope after MDA modification of proteins in atherosclerosis. Free Radic. Biol. Med..

[17] Chang M.K., Bergmark C., Laurila A., Horkko S., Han K.H., Friedman P. (1999). Monoclonal antibodies against oxidized low-density lipoprotein bind to apoptotic cells and inhibit their phagocytosis by elicited macrophages: evidence that oxidation-specific epitopes mediate macrophage recognition. Proc. Natl. Acad. Sci. U. S. A..

[18] Gonen A., Hansen L.F., Turner W.W., Montano E.N., Que X., Rafia A. (2014). Atheroprotective immunization with malondialdehyde-modified LDL is hapten specific and dependent on advanced MDA adducts: implications for development of an atheroprotective vaccine. J. Lipid Res..

[19] Weismann D., Hartvigsen K., Lauer N., Bennett K.L., Scholl H.P., Charbel Issa P. (2011). Complement factor H binds malondialdehyde epitopes and protects from oxidative stress. Nature.

[20] Rudnick R.B., Chen Q., Stea E.D., Hartmann A., Papac-Milicevic N., Person F. (2018). FHR5 binds to laminins, uses separate C3b and surface-binding sites, and activates complement on malondialdehyde-acetaldehyde surfaces. J. Immunol..

[21] Veneskoski M., Turunen S.P., Kummu O., Nissinen A., Rannikko S., Levonen A.L. (2011). Specific recognition of malondialdehyde and malondialdehyde acetaldehyde adducts on oxidized LDL and apoptotic cells by complement anaphylatoxin C3a. Free Radic. Biol. Med..

[22] Yeh F.L., Wang Y., Tom I., Gonzalez L.C., Sheng M. (2016). TREM2 binds to apolipoproteins, including APOE and CLU/APOJ, and thereby facilitates uptake of amyloid-beta by microglia. Neuron.

[23] Yin C., Ackermann S., Ma Z., Mohanta S.K., Zhang C., Li Y. (2019). ApoE attenuates unresolvable inflammation by complex formation with activated C1q. Nat. Med..

[24] Busch C.J., Hendrikx T., Weismann D., Jackel S., Walenbergh S.M., Rendeiro A.F. (2017). Malondialdehyde epitopes are sterile mediators of hepatic inflammation in hypercholesterolemic mice. Hepatology.

[25] Palinski W., Ord V.A., Plump A.S., Breslow J.L., Steinberg D., Witztum J.L. (1994). ApoE-deficient mice are a model of lipoprotein oxidation in atherogenesis. Demonstration of oxidation-specific epitopes in lesions and high titers of autoantibodies to malondialdehyde-lysine in serum. Arterioscler. Thromb..

[26] Chou M.Y., Fogelstrand L., Hartvigsen K., Hansen L.F., Woelkers D., Shaw P.X. (2009). Oxidation-specific epitopes are dominant targets of innate natural antibodies in mice and humans. J. Clin. Invest..

[27] Tuominen A., Miller Y.I., Hansen L.F., Kesaniemi Y.A., Witztum J.L., Horkko S. (2006). A natural antibody to oxidized cardiolipin binds to oxidized low-density lipoprotein, apoptotic cells, and atherosclerotic lesions. Arterioscler. Thromb. Vasc. Biol..

[28] Ondracek A.S., Afonyushkin T., Aszlan A., Taqi S., Koller T., Artner T. (2025). Malondialdehyde-specific natural IgM inhibit NETosis triggered by culprit site-derived extracellular vesicles from myocardial infarction patients. Eur. Heart J..

[29] Tenner A.J., Lesavre P.H., Cooper N.R. (1981). Purification and radiolabeling of human C1q. J. Immunol..

[30] Dahlback B. (1983). Purification of human C4b-binding protein and formation of its complex with vitamin K-dependent protein S. Biochem. J..

[31] Blom A.M., Kask L., Dahlback B. (2001). Structural requirements for the complement regulatory activities of C4BP. J. Biol. Chem..

[32] Nilsson S.C., Karpman D., Vaziri-Sani F., Kristoffersson A.C., Salomon R., Provot F. (2007). A mutation in factor I that is associated with atypical hemolytic uremic syndrome does not affect the function of factor I in complement regulation. Mol. Immunol..

[33] Greenwood F.C., Hunter W.M., Glover J.S. (1963). The preparation of I-131-Labelled human growth hormone of high specific radioactivity. Biochem. J..

[34] Andersson M., Hanson A., Englund G., Dahlback B. (1991). Inhibition of complement components C3 and C4 by cadralazine and its active metabolite. Eur. J. Clin. Pharmacol..

[35] Wenderfer S.E., Soimo K., Wetsel R.A., Braun M.C. (2007). Analysis of C4 and the C4 binding protein in the MRL/lpr mouse. Arthritis. Res. Ther..

[36] Bertheloot D., Latz E., Franklin B.S. (2021). Necroptosis, pyroptosis and apoptosis: an intricate game of cell death. Cell Mol. Immunol..

[37] Song X., Xie Y., Kang R., Hou W., Sun X., Epperly M.W. (2016). FANCD2 protects against bone marrow injury from ferroptosis. Biochem. Biophys. Res. Commun..

[38] Feng H., Schorpp K., Jin J., Yozwiak C.E., Hoffstrom B.G., Decker A.M. (2020). Transferrin receptor is a specific ferroptosis marker. Cell Rep..

[39] Zhang Y., Tan H., Daniels J.D., Zandkarimi F., Liu H., Brown L.M. (2019). Imidazole ketone erastin induces ferroptosis and slows tumor growth in a mouse lymphoma model. Cell Chem. Biol..

[40] Blom A.M., Webb J., Villoutreix B.O., Dahlback B. (1999). A cluster of positively charged amino acids in the C4BP alpha-chain is crucial for C4b binding and factor I cofactor function. J. Biol. Chem..

[41] Gigli I., Fujita T., Nussenzweig V. (1979). Modulation of the classical pathway C3 convertase by plasma proteins C4 binding protein and C3b inactivator. Proc. Natl. Acad. Sci. U. S. A..

[42] Webb J.H., Blom A.M., Dahlback B. (2002). Vitamin K-dependent protein S localizing complement regulator C4b-binding protein to the surface of apoptotic cells. J. Immunol..

[43] Kristensen T., Ogata R.T., Chung L.P., Reid K.B., Tack B.F. (1987). cDNA structure of murine C4b-binding protein, a regulatory component of the serum complement system. Biochemistry.

[44] Barnum S.R., Kristensen T., Chaplin D.D., Seldin M.F., Tack B.F. (1989). Molecular analysis of the murine C4b-binding protein gene. Chromosome assignment and partial gene organization. Biochemistry.

[45] Rodriguez de Cordoba S., Perez-Blas M., Ramos-Ruiz R., Sanchez-Corral P., Pardo-Manuel de Villena F., Rey-Campos J. (1994). The gene coding for the beta-chain of C4b-binding protein (C4BPB) has become a pseudogene in the mouse. Genomics.

[46] Alic L., Binder C.J., Papac-Milicevic N. (2022). The OSE complotype and its clinical potential. Front. Immunol..

[47] Zhang Y., Chen X., Gueydan C., Han J. (2018). Plasma membrane changes during programmed cell deaths. Cell Res..

[48] Elliott M.R., Ravichandran K.S. (2016). The dynamics of apoptotic cell clearance. Dev. Cell.

[49] Suurmond J., Diamond B. (2015). Autoantibodies in systemic autoimmune diseases: specificity and pathogenicity. J. Clin. Invest..

[50] Li J., Cao F., Yin H.L., Huang Z.J., Lin Z.T., Mao N. (2020). Ferroptosis: past, present and future. Cell Death Dis..

[51] Samson A.L., Zhang Y., Geoghegan N.D., Gavin X.J., Davies K.A., Mlodzianoski M.J. (2020). MLKL trafficking and accumulation at the plasma membrane control the kinetics and threshold for necroptosis. Nat. Commun..

[52] Rogers C., Fernandes-Alnemri T., Mayes L., Alnemri D., Cingolani G., Alnemri E.S. (2017). Cleavage of DFNA5 by caspase-3 during apoptosis mediates progression to secondary necrotic/pyroptotic cell death. Nat. Commun..

[53] Taleb A., Willeit P., Amir S., Perkmann T., Kozma M.O., Watzenbock M.L. (2023). High immunoglobulin-M levels to oxidation-specific epitopes are associated with lower risk of acute myocardial infarction. J. Lipid Res..

[54] Karvonen J., Paivansalo M., Kesaniemi Y.A., Horkko S. (2003). Immunoglobulin M type of autoantibodies to oxidized low-density lipoprotein has an inverse relation to carotid artery atherosclerosis. Circulation.

[55] Tsimikas S., Willeit P., Willeit J., Santer P., Mayr M., Xu Q. (2012). Oxidation-specific biomarkers, prospective 15-year cardiovascular and stroke outcomes, and net reclassification of cardiovascular events. J. Am. Coll. Cardiol..

[56] Ravandi A., Boekholdt S.M., Mallat Z., Talmud P.J., Kastelein J.J., Wareham N.J. (2011). Relationship of IgG and IgM autoantibodies and immune complexes to oxidized LDL with markers of oxidation and inflammation and cardiovascular events: results from the EPIC-Norfolk study. J. Lipid Res..

[57] van den Berg V.J., Haskard D.O., Fedorowski A., Hartley A., Kardys I., Caga-Anan M. (2018). IgM anti-malondialdehyde low density lipoprotein antibody levels indicate coronary heart disease and necrotic core characteristics in the Nordic Diltiazem (NORDIL) study and the integrated imaging and biomarker study 3 (IBIS-3). EBioMedicine.

[58] Gruber S., Hendrikx T., Tsiantoulas D., Ozsvar-Kozma M., Goderle L., Mallat Z. (2016). Sialic acid-binding immunoglobulin-like lectin G promotes atherosclerosis and liver inflammation by suppressing the protective functions of B-1 cells. Cell Rep..

[59] Obermayer G., Afonyushkin T., Goderle L., Puhm F., Schrottmaier W., Taqi S. (2021). Natural IgM antibodies inhibit microvesicle-driven coagulation and thrombosis. Blood.

[60] Garcia de Frutos P., Alim R.I., Hardig Y., Zoller B., Dahlback B. (1994). Differential regulation of alpha and beta chains of C4b-binding protein during acute-phase response resulting in stable plasma levels of free anticoagulant protein S. Blood.

[61] Jensen M.L., Honore C., Hummelshoj T., Hansen B.E., Madsen H.O., Garred P. (2007). Ficolin-2 recognizes DNA and participates in the clearance of dying host cells. Mol. Immunol..

[62] Ogden C.A., deCathelineau A., Hoffmann P.R., Bratton D., Ghebrehiwet B., Fadok V.A. (2001). C1q and mannose binding lectin engagement of cell surface calreticulin and CD91 initiates macropinocytosis and uptake of apoptotic cells. J. Exp. Med..

[63] Palaniyar N., Nadesalingam J., Clark H., Shih M.J., Dodds A.W., Reid K.B. (2004). Nucleic acid is a novel ligand for innate, immune pattern recognition collectins surfactant proteins A and D and mannose-binding lectin. J. Biol. Chem..

[64] Garred P., Genster N., Pilely K., Bayarri-Olmos R., Rosbjerg A., Ma Y.J. (2016). A journey through the lectin pathway of complement-MBL and beyond. Immunol. Rev..

[65] Baudino L., Sardini A., Ruseva M.M., Fossati-Jimack L., Cook H.T., Scott D. (2014). C3 opsonization regulates endocytic handling of apoptotic cells resulting in enhanced T-cell responses to cargo-derived antigens. Proc. Natl. Acad. Sci. U. S. A..

[66] Bierschenk D., Papac-Milicevic N., Bresch I.P., Kovacic V., Bettoni S., Dziedzic M. (2023). C4b-binding protein inhibits particulate- and crystalline-induced NLRP3 inflammasome activation. Front. Immunol..

[67] King B.C., Kulak K., Colineau L., Blom A.M. (2021). Outside in: roles of complement in autophagy. Br. J. Pharmacol..

[68] Amarilyo G., Verbovetski I., Atallah M., Grau A., Wiser G., Gil O. (2010). iC3b-opsonized apoptotic cells mediate a distinct anti-inflammatory response and transcriptional NF-kappaB-dependent blockade. Eur. J. Immunol..

[69] Clarke E.V., Weist B.M., Walsh C.M., Tenner A.J. (2015). Complement protein C1q bound to apoptotic cells suppresses human macrophage and dendritic cell-mediated Th17 and Th1 T cell subset proliferation. J. Leukoc. Biol..

[70] Kask L., Trouw L.A., Dahlback B., Blom A.M. (2004). The C4b-binding protein-protein S complex inhibits the phagocytosis of apoptotic cells. J. Biol. Chem..

[71] Olivar R., Luque A., Naranjo-Gomez M., Quer J., Garcia de Frutos P., Borras F.E. (2013). The alpha7beta0 isoform of the complement regulator C4b-binding protein induces a semimature, anti-inflammatory state in dendritic cells. J. Immunol..

[72] Serrano I., Luque A., Mitjavila F., Blom A.M., Rodriguez de Cordoba S., Vega M.C. (2022). The hidden side of complement regulator C4BP: dissection and evaluation of its immunomodulatory activity. Front. Immunol..

[73] Kulak K., Westermark G.T., Papac-Milicevic N., Renstrom E., Blom A.M., King B.C. (2017). The human serum protein C4b-binding protein inhibits pancreatic IAPP-induced inflammasome activation. Diabetologia.

[74] Kiss M.G., Papac-Milicevic N., Porsch F., Tsiantoulas D., Hendrikx T., Takaoka M. (2023). Cell-autonomous regulation of complement C3 by factor H limits macrophage efferocytosis and exacerbates atherosclerosis. Immunity.

