# Supplementary material for: Malondialdehyde epitopes exposed on dying cells are potent modulators of complement activity and efferocytosis
Source: J Lipid Res. 2026 Jul 9;67(8):101100. doi: 10.1016/j.jlr.2026.101100 (PMC13520893; doi:10.1016/j.jlr.2026.101100)
Supplement: Supplementary file 1 [file mmc1.docx]

**Supplemental Information**

**Supplemental Figure 1.** Flow cytometry-based assays for determining the presence of MDA-epitopes on the surface of apoptotic cells. Representative viability plots are divided into quadrants based on 7AAD and Annexin V positivity, while MDA^+^ cells are distinguished by color (blue, orange , tan; left panel). Corresponding quantifications of IC^+^ or MDA^+^ populations within Annexin V^+^ cells (middle panel), and percentage of Annexin V^+^ cells within total cells (right panel) are shown. A.) Absence of MDA^+^ signal when isotype control (IC) antibody was used for staining of Jurkat T cells irradiated with different UVC dose-curve treatments. B.) The same pattern of the appearance of MDA-epitopes on Jurkat T-cells was observed when MDA-epitopes are detected with NA17 antibody after induction of apoptosis by UVC. C.) Time course for appearance of MDA-epitopes detected by LR04 antibody on Jurkat T-cells upon at 4, 12 and 24 hours after induction of apoptosis by 2µM staurosporine (Stau) or after treatment with DMSO vehicle control. D.) Induction of extrinsic apoptotic pathway by anti-CD95 or control isotype antibody (IC) and appearance of MDA-epitopes on Jurkat T cells. Shown is the percentage of MDA^+^ cells from the total cells. E.) Time course for appearance of MDA-epitopes on primary murine thymocytes 4, 12 and 24 hours after UVC irradiation with 100mJ/cm^2^.

Bars represent mean ± SD values of triplicate determinations pooled from two independent experiments. Statistical differences were tested by one-way ANOVA, with Tukey’s multiple comparisons (*p-value ≤ 0.05, ***p-value ≤ 0.001, ****p-value ≤ 0.0001).

**Supplemental Figure 2**. A.) Colocalization of MDA-epitopes (LR04 signal=green, first column of images) with cellular membrane (WGA=red, second column) determined by confocal microscopy. Nucleus was counterstained with DAPI (blue, third column). Yellow fluorescence reveals the potential colocalization of MDA^++^ signal (high abundance) and cellular membrane (right panel, merged image, fourth column) (scale bar represents 10µm, LSM-700, objective 63x). Staining of apoptotic cells with isotype control IgM and WGA served as a negative control (lower panel) B.) Graphical representation of colocalization analysis based on Pearson's correlation between IC or MDA^++^ signals and cellular membrane detected by WGA signal. Each spot represents a single analyzed image. C.) Graphical representation of colocalization analysis based on Manders' coefficient (fraction of overlap between MDA-WGA channels) within MDA^++^ images. Data are represented as a mean. Each spot represents a single analyzed image. Statistical differences were tested by one-way ANOVA, with Tukey’s multiple comparisons (****p-value ≤ 0.0001).

**Supplemental Figure 3.** A.) FACS-based assay for determining the presence of MDA-epitopes on ferroptotic cells. Representative plots are divided into quadrants based on 7AAD and MDA positivity (NA17) (left), and corresponding quantifications (right) are shown. B.) FACS-based assay for determining the presence of signal when IC antibody was used for staining ferroptotic cells. Representative plots are divided into quadrants based on 7AAD and APC^+^ positivity (IC) (left), and corresponding quantifications (right) are shown. C.) Confocal images of MDA-epitopes (NA17 signal=green) detected by NA17 antibody and present on ferroptotic cells (DAPI= blue) (upper panel). No signal was observed when ferroptotic cells were stained with IC antibody (lower panel) (scale bar represents 20µm, LSM780, objective 20x). D.) FACS-based assay for determining the presence of signal when IC antibody was used for staining necroptotic FADD-/- Jurkat cells. Representative plots are divided into quadrants based on 7AAD and APC^+^ positivity (left), and corresponding quantifications (right) are shown. E.) Detection of appearance of MDA-epitopes detected by NA17 antibody (NA17 signal= green) on the surface of necroptotic FADD-/- Jurkats cells (DAPI= blue) (scale bar represents 10µm, LSM780, objective 40x). Necroptosis was induced by TNFα and inhibited by Necrostatin-1 (Nec-1) or by Necrosulfonamide (NSA). Bars represent mean ± SD values of triplicate determinations pooled from two or three independent experiments. Statistical differences were tested by one-way ANOVA, with Tukey’s multiple comparisons (***p-value ≤ 0.001).

**Supplemental Figure 4.** A. & B.) ELISA for detection of binding of purified C1q to various OSE modifications of BSA and LDL, such as 4-hydroxynonenal BSA (4-HNE-BSA), MDA-BSA, MAA-BSA, phosphocholine-BSA (PC-BSA), MDA-LDL, MAA-LDL, and CuOx-LDL. A.) Binding of plasma-purified C1q protein to MAA-BSA, MDA-BSA, and onto coated IgG (positive control). B.) Binding of purified C1q protein to MAA-LDL, MDA-LDL, and to coated IgG (positive control). C.) ELISA for monitoring activation of the alternative pathway was by measuring the deposition of C9. D. & E.) ELISA for the detection of binding of C3met to various OSE modifications of BSA and LDL. D.) Binding of C3met protein to MAA-BSA, MDA-BSA, and onto coated complement factor H (FH) (positive control). E.) Binding of C3met to MAA-LDL, MDA-LDL, and onto coated FH (positive control). F.) ELISAs for measuring deposition of C3b from normal human serum (+NHS) or heat-inactivated serum (+HI-NHS) to coated zymosan, MAA-BSA, sham BSA, or naïve BSA. G.) ELISAs for measuring deposition of C3b from Factor B-depleted human serum (-FB) or FB-depleted serum with the restored amount of FB (+FB) to coated zymosan, MAA-BSA, sham BSA, or naïve BSA. Values are expressed as mean ± SD.

**Supplemental Figure 5**. A.) ELISA for characterization of the nature of binding between C4BP variants (α7β1+PS and α6β0) and MAA-BSA in the presence of increasing concentration of NaCl. Values are expressed as mean± SEM. Statistical differences were tested by one-way ANOVA, with Tukey’s multiple comparisons (***p-value ≤ 0.001, ****p-value ≤ 0.0001). B.) C4b degradation assay in a plate. The recombinant C4BP was left to bind possible ligands coated on the wells, and later it was incubated with recombinant factor I and trace amounts of ^125^I-labeled C4b. The amount of generated C4d fragments was determined by SDS-PAGE. C.) Quantification of C4d fragments bands represented as C4d/α-chain ratio. Statistical differences were tested by two-way ANOVA, with Bonferroni’s multiple comparisons (****p-value ≤ 0.0001). Values are expressed as mean± SD.

**Supplemental Figure 6.** A.) Quantifications of Annexin V positivity from total cells observed in FACS-based assay on MDA^high^ (H) and MDA^low^ (L) cellular fractions. B.) Quantification of opsonization by C4b of MDA^high^ (H) and MDA^low^ (L) dying cells fractions by FACS detected from NHS (0.5%, 1%, or 2%). C.) Binding of purified various C4BP variants (α7β1+PS, α7β1, α7β0, α6β0) to MDA^high^ (H) (no border on bars, left) and MDA^low^ (L) (bordered bars, right) cells measured in FACS-based assay. Representative plots are divided into quadrants based on 7AAD, and Annexin V positivity (left) and corresponding quantifications (upper panel on right) are shown. Quantifications of Annexin V positivity observed in FACS-based assay were binding of different C4BP variants (α7β1+PS, α7β1, α7β0, α6β0) to MDA^high^ (H) and MDA^low^ (L) cellular fractions were tested in a buffer containing physiological levels of Ca^2+^(lower panel on right) D.) FACS-based assay for determining the recruitment of 6α0β C4BP on apoptotic and necroptotic FADD-/- Jurkat cells. Representative plots are divided into quadrants based on 7AAD and C4BP APC positivity. Bars represent mean ± SD values of triplicate determinations pooled from two or three independent experiments. Statistical differences were tested by one-way ANOVA, with Tukey’s multiple comparisons (**p-value ≤ 0.01, ****p-value ≤ 0.0001).

**Supplemental Figure 7.** A.) FACS-based assays for determining the recruitment of mC4BP from 2,5 and 5% NMS on the surface of viable (v) and apoptotic cells (a) human Jurkat T cells. Representative viability plots (left panel) are divided into quadrants based on 7AAD and Annexin V positivity, while MDA^+^ cells are distinguished by color (blue & orange, depending on NMS percentage). Corresponding quantifications of MDA^+^ populations within total cells (right panel) are shown. B.) Percentage of CD11b^+^/F480^+^ macrophages in efferocytosis experiment (Figure 5F) determined by FACS-based assay. Bars represent mean ± SD values of triplicate determinations pooled from two or three independent experiments. Statistical differences were tested by one-way ANOVA, with Tukey’s multiple comparisons (**p-value ≤ 0.01, ****p-value ≤ 0.0001). C.) Schematic representation of the proposed consequences of classical complement pathway activation and its regulation on MDA-bearing surfaces. MDA-epitopes trigger classical complement activation via IgM and/or C1q binding, resulting in the formation of the C3 convertase (C4bC2a), which can further propagate the complement cascade. In the presence of C4bp, which also binds to MDA-epitopes, the formation of the C3 convertase is inhibited, thereby preventing the generation of pro-inflammatory mediators in this context.
